# Low-field MRI: Clinical promise and challenges

**DOI:** 10.1002/jmri.28408

**Published:** 2022-09-19

**Authors:** Thomas Campbell Arnold, Colbey W. Freeman, Brian Litt, Joel M Stein

**Affiliations:** 1 Department of Bioengineering, School of Engineering & Applied Science, University of Pennsylvania, Philadelphia, Pennsylvania, USA; 2 Center for Neuroengineering and Therapeutics, University of Pennsylvania, Philadelphia, Pennsylvania, USA; 3 Department of Radiology, Perelman School of Medicine, University of Pennsylvania, Philadelphia, Pennsylvania, USA; 4 Department of Neurology, Perelman School of Medicine, University of Pennsylvania, Philadelphia, Pennsylvania, USA

## Abstract

Modern MRI scanners have trended toward higher field strengths to maximize signal and resolution while minimizing scan time. However, high-field devices remain expensive to install and operate, making them scarce outside of high-income countries and major population centers. Low-field strength scanners have drawn renewed academic, industry, and philanthropic interest due to advantages that could dramatically increase imaging access, including lower cost and portability. Nevertheless, low-field MRI still faces inherent limitations in image quality that come with decreased signal. In this article, we review advantages and disadvantages of low-field MRI scanners, describe hardware and software innovations that accentuate advantages and mitigate disadvantages, and consider clinical applications for a new generation of low-field devices. In our review, we explore how these devices are being or could be used for high acuity brain imaging, outpatient neuroimaging, MRI-guided procedures, pediatric imaging, and musculoskeletal imaging. Challenges for their successful clinical translation include selecting and validating appropriate use cases, integrating with standards of care in high resource settings, expanding options with actionable information in low resource settings, and facilitating health care providers and clinical practice in new ways. By embracing both the promise and challenges of low-field MRI, clinicians and researchers have an opportunity to transform medical care for patients around the world.

Magnetic resonance imaging (MRI) is a mainstay of modern medicine and has led to significant advances in basic science and clinical patient care. MRI has superior soft tissue contrast and provides definitive diagnostic information throughout the body, particularly exceling in neuroimaging and musculoskeletal applications. MRI is widely utilized in high-income countries (HICs), with 1.9 scans annually per 10 American Medicare enrollees.^1^ However, high costs and technical barriers have limited adoption in low- and middle-income countries (LMICs).^2^ Worldwide, approximately 90% of people lack access to MRI,^3^ while two-thirds lack even basic medical imaging.^4,5^ Even in HICs, MRI is unavailable in rural areas and to patients with disability or device constraints.^6,7^ Recent advances in lower-field strength MRI offer potential solutions, with less expensive and portable devices. However, lower-field MRI still faces significant challenges, and it remains to be seen how newer devices will be deployed clinically.

Most MRI units today use high-field strength, cryogenically-cooled, superconducting magnets, though low-field permanent and resistive magnet designs have existed throughout MRI’s history. Permanent magnets offer decreased cost and siting requirements but achieve lower magnetic field strengths, which impacts the signal-to-noise ratio (SNR) achieved per unit time during scanning. Lower-field devices typically acquire lower resolution images to maintain clinically feasible scan times. Recent academic and industry efforts seek to leverage lower-field strength advantages including lower cost, smaller device footprints, and fewer safety concerns while mitigating inherent disadvantages that contribute to lower image quality (Table 1). To balance these factors, lower-field approaches are increasingly tailored to specific clinical questions and contexts. Selecting and validating appropriate use cases remains paramount.

In this review, we explore both the clinical promise and challenges of lower-field MRI. We describe hardware and software advances as well as financial and practical considerations related to lower-field device adoption. Next, we discuss five clinical domains where lower-field MRI offers clinical promise: high acuity brain imaging, outpatient neuroimaging, MRI-guided procedures, pediatric imaging, and musculoskeletal imaging. In each section, we provide a literature overview, examples of low-field use, and a discussion of how new devices can integrate with current care standards. Although we cannot cover all use cases, our goal is to convey the technology’s potential impact and stimulate further clinical translation.

## Hardware & Software Advances

**FIGURE 1:**
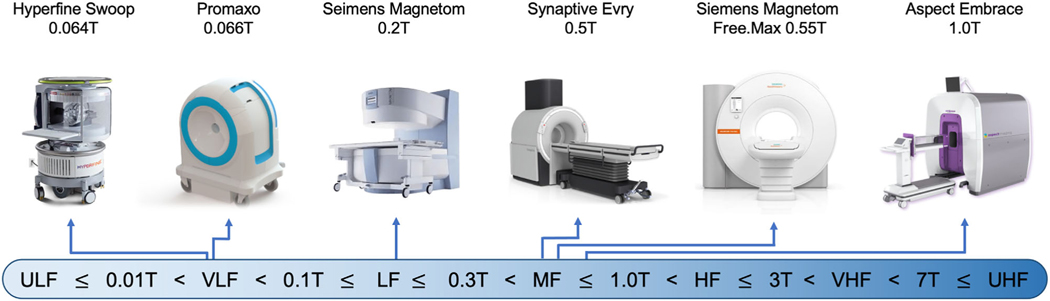
Defining “low-field” MRI. This figure defines how scanners at difference field strengths will be categorized in the article, with the following boundaries illustrated on the bottom: Ultra-low-field (ULF) ≤ 0.01T < very-low-field (VLF) < 0.1T ≤ low-field (LF) ≤ 0.3T < mid-field (MF) ≤ 1.0T < high-field (HF) ≤ 3T < very-high-field (VHF) < 7T ≤ ultra-high-field (UHF). Select commercially available scanners with a field strength of 1 Tesla or lower are illustrated on the top. Scanners are not to scale. Scanner images are copyright of the respective manufacturers. Images used with permission or in accordance with manufacturer pol

The definition of “low-field” varies, sometimes referring to anything below 1.5T while other times indicating a narrow band between 0.01T and 0.1T. For this review, we refer to devices using the distinctions in Fig. 1. We use “lower-field” to broadly describe devices below 1.5T and “higher-field” for 1.5T and above. New levels of distinction will continue to be developed to better communicate; however, we can obviate confusion by defining terminology in our work or developing standards through professional societies, such as the International Society of Magnetic Resonance Medicine.

**FIGURE 2:**
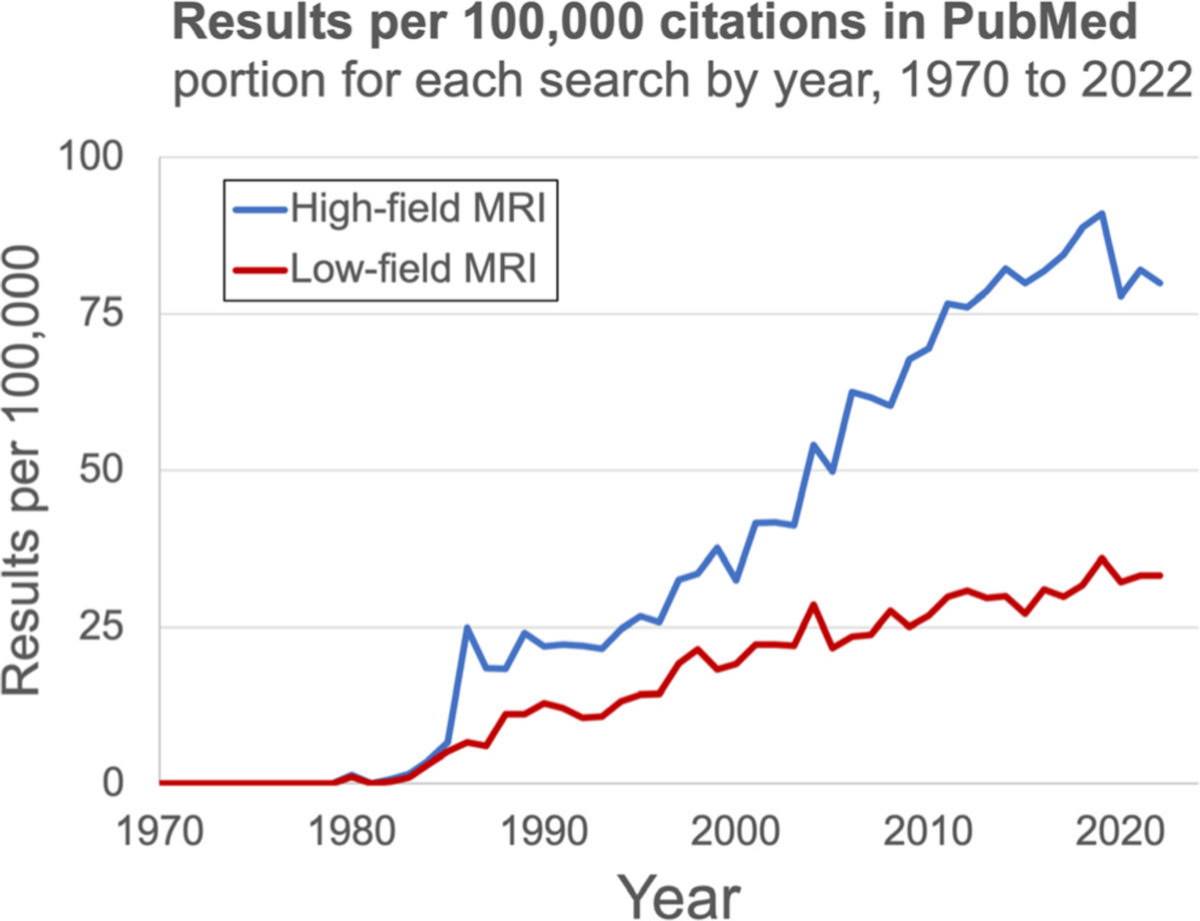
Research interest in low-field. The relative number of PubMed citations^8^ for high-field MRI (blue) and low-field MRI (red) have been diverging in recent decades, reflecting the dominance of high-field scanners.

The distinction between low-field and high-field MRI appeared during MRI’s infancy in the 1980s (Fig. 2).^8^ A citation gap emerged after 1985, when the first 1.5T scanners were introduced.^9^ The gap grew throughout the 1990s and widened significantly in the early 2000s, when 1.5T scanners became the clinical standard.^10^ High-field scanners gained a dominant market share because of their higher SNR per unit time, which permits faster imaging, higher resolution, greater contrast sensitivity, and more advanced sequences.^11^ Commercial lower-field devices have remained available over this time period, but many have been relegated to niche use cases or discontinued. Nevertheless, renewed commercial interest has led to FDA clearance of several lower-field systems since 2018, including the 0.064T Hyperfine Swoop head scanner, 0.066T Promaxo prostate scanner, 0.5T Synaptive Evry intraoperative scanner, 0.55T Siemens Magnetom Free.Max general purpose scanner, and 1T Aspect Embrace neonatal scanner. While high-field devices won market dominance based on higher image quality, two primary factors are driving this recent lower-field renaissance: 1) lower scanner costs and 2) technological innovations resulting in image quality improvements.^11^

Medical care costs in the United States have risen dramatically, with medical imaging contributing significantly. High-field MRI devices are expensive, and their cost has increased over time. The largest component of MRI device cost is the magnet, with total high-field device cost being roughly 1 million USD per Tesla.^12^ Lower-field strength devices offer significant device cost savings. Although lower-field MRI is associated with lower image quality, it is actually SNR per unit time that is proportionate to magnetic field strength. Stronger magnets reduce the time necessary to achieve a certain level of sensitivity.^11^ Given sufficient time, lower magnetic fields can produce high-SNR images of diagnostic quality; however, patient tolerance and clinical expediency place practical constraints on acquisition times. Recent software and hardware advances have improved image quality obtained per unit time, making imaging at lower-field strengths within clinically relevant quality and timeframe standards feasible.

Technological developments spurring interest in lower-field devices include hardware improvements (eg improved magnet, gradient, and coil designs) and software developments (eg deep learning reconstruction and post-processing). Multiple research groups have pioneered the development of newer lower field devices, including the ultra-low-field 0.0065T electromagnet scanner at MGH,^12^ the 0.08T and 0.05T Halbach array devices respectively at MGH and the University of Leiden,^13,14^ a fast field cycling scanner at the University of Aberdeen that can operate between 50μT and 0.2T,^15^ and the 0.05T and 0.055T permanent dipole systems at Chongqing University and the University of Hong Kong, respectively.^16,17^ A common theme is design simplification to facilitate scanner production, maintenance, and operation in low-resource settings.^18^ Additionally, reduced weight and siting requirements enable some devices to be portable. At lower-field strengths, coil noise is dominant, leading researchers to optimize wire diameter, spacing, and windings in low-cost, 3D-printed head coils.^12^ Portable systems must have lightweight radiofrequency (RF) shielding. Researchers eliminated bulky shielding by using passive coils to predict and remove electromagnetic noise.^16,19^ Another trend is reduced reliance on gradient coils, which require high amounts of power. Cooley et al designed a cylindrical Halbach array, a scanner composed of multiple small permanent magnets, with optimized magnet placement resulting in a built-in readout field gradient with minimal stray flux.^13^ Importantly, this eliminates one gradient system, lowering the devices power and cooling requirements. Additionally, they leveraged a rotating scanner bore to collect 2D images without any gradient coils, thereby permitting silent imaging.^20^ Another approach has been to step-down high-field systems to operate at lower-field strengths while maintaining state-of-the-art commercial gradients and coils.^21,22^

Software advances have been facilitated by deep learning advancements, increased graphics processing unit availability, and the open-source movement. With decreased SNR per unit time, lower-field strengths accentuate the trade-off between resolution and scan time. Lower-field strength scanners can leverage reduced specific absorption rates (eg Transmit Array Spatial Encoding, shorter RF pulses, longer spin echo trains) and SNR efficient acquisition strategies (eg bSSFP, MR fingerprinting, long readout spiral imaging), to maximize image quality per unit time.^3,23^ Additionally, to reduce scan times researchers sought rapid imaging methods, such as sensor space subsampling; however, this results in noise and image artifacts after conventional reconstruction. Recently, compressed sensing and deep learning have enabled reconstruction from a smaller subset of k-space.^24,25^ Deep learning reconstruction methods use neural networks to learn robust transformation mappings from sensor space to the image domain. Image postprocessing has also benefited from deep learning, with applications in super-resolution,^26–28^ segmentation,^29^ simulation,^30^ denoising,^31^ and artifact rejection.^32^ However, analytical software development typically lags hardware advances. It may take several years for some software commonly used at high-field to be adapted to low-field scanners. The low-field research community has engaged with the open-source movement, most notably through the Open Source Imaging Initiative (OSI^2^: opensourceimaging.org),^33,34^ which may facilitate faster development of both hardware and software applications.^14,35,36^

While we must acknowledge the hardware and software innovations that have led to the lower-field device resurgance, a full discussion is beyond the scope of this review. For more information, we recommend Wald et al^37^ & Marques et al.^3^

## Financial and Practical Considerations

Lower-field MRI adoption requires an understanding of how device costs and implementation differs from traditional high-field scanners. Lower-field strength devices typically cost less and have reduced siting requirements, enabling them to be used for novel applications and in new settings. As MRI is expanded into new patient populations and care environments, it is crucial that researchers, device manufacturers, and care providers understand the relevant constraints in these settings. Here, we review practical and financial considerations that should guide appropriate clinical application selection.

One large advantage of lower-field devices is reduced siting requirements compared to traditional high-field systems (Table 1). High-field devices are large, usually weighing over 5 tons and requiring two dedicated rooms with reinforced flooring and RF shielding.^38^ Most high-field scanners use superconducting magnets, which require additional high-power infrastructure and a quench pipe for cryogenic cooling. These devices are sensitive to vibrations and nearby ferromagnetic objects (eg ambulances, cars, trains). By contrast, many lower-field strength devices weigh less, with several scanners reported between 0.05 and 0.25 tons.^36^ They require less or no RF shielding.^16,19^ Low-field devices are often permanent magnets, reducing overall power demands and eliminating cryogenic cooling. Additionally, the 5 gauss safety line scales with magnetic field strength, enabling lower-field devices to be in closer proximity to other scanners, medical equipment, and ferromagnetic objects.^39^ The lower siting requirements significantly reduces installation costs and overall device footprint, facilitating portability in some cases.

MRI resources tend to be concentrated in population centers, resulting in reduced imaging access in rural areas and introducing sampling bias into research studies.^40^ Tractor trailers have been retrofit with 1.5T magnets to increase access. Mobile scanners enable device cost sharing between hospitals and permit imaging in restricted populations.^41^ However, these devices cost millions of dollars and have complicated infrastructure, limiting their deployment. Recently, research groups have retrofit vans with lower-field devices. Nakagomi et al placed a 0.2T magnet in a minivan for mobile extremity imaging.^42^ They envisioned deploying the device to sporting events or areas without MRI access. Deoni et al retrofit a Ford Transit van with a 0.064T magnet and demonstrated neuroimaging in pediatric and adult patients at their homes.^43^ The estimated project cost was 110,000 USD, a fraction of the cost to purchase a mobile 1.5T tractor trailer.

Increased healthcare costs in the United States have led to a critical evaluation of medical imaging expenditure.^44^ In addition to optimizing current practices,^45^ increased reliance on lower-field devices may offer a cost-effective means of enhancing MRI value. Japan, which has the highest concentration of MRI devices worldwide, has capped MRI reimbursement rates.^3,46^ This led to widespread adoption of low-field devices, which offer lower cost per examination and thus increased profitability. While there are undoubtedly cases when high-field MRI is more clinically appropriate, it may be reasonable to adopt a similar approach to Japan, where high-field scanners are concentrated in healthcare centers and mid-to-low-field devices are more widely available. While SNR per unit time is proportional to field strength, this may not be the best metric for determining how much value different MRI systems contribute to diagnostic accuracy, patient outcomes, and societal benefit.^47^ Low-field devices may allow patient triage and reduce scheduling demands on high-field scanners, resulting in decreased diagnostic delays and increased patient satisfaction.^48,49^

While low-field devices may augment standard-of-care (SOC) imaging in HICs, they will likely play a more impactful role in LMICs. In 2016, an estimated 84 MRI units serviced West Africa, an area of over 370 million people.^50^ For comparison, in 2019, the United States had an estimated 13,000 devices to service ~330 million people. Low-field devices already play a dominant role in MRI services in West African countries, with the majority (77.6%) of devices in Nigeria being low-field strength (<0.3T).^38,50^ Neuroimaging was the primary application, with one center reporting over 90% of studies requested were for brain (49.9%) or spine (45.6%) imaging.^51^ However, the average MRI cost was ~500 USD and services are typically paid by patients out-of-pocket, making even low-field scanners beyond the reach of a significant portion of the population.^51^

Increased geographical and financial access has been a primary motivator for ultra-low-field and very-low-field system development.^2^ This includes devices targeting pediatric hydrocephalus, which has a high prevalence in Africa.^52,53^ More targeted systems may be cheaper to produce and service, allowing for lower out-of-pocket costs. Ogbole et al noted that lack of technical support or service materials caused significant scanner downtime.^51^ When designing devices for LMICs, special consideration should be given to available resources and expertise.^54,55^ Additionally, many LMICs have a dearth of radiologists and radiographers.^56^ Remote readers or automated algorithms may provide diagnostic support, allowing countries to stretch scarce resources.^57^ For more details on imaging accessibility, the authors recommend Geethanath et al.^2^

Equally important to proper device design is appropriate and equitable introduction of devices into society. Recently, working groups of researchers, clinicians, MR vendors, and local stakeholders have convened to provide guidelines and address key ethical, legal, and social questions surrounding portable MRI.^58,59^ Continued working group engagement will be essential for providing updated recommendations as new hardware and software are released.

The remainder of this review focuses on potential clinical applications of newer lower-field MRI devices. While we focus on neuroimaging and musculoskeletal applications, lower-field MRI offers opportunities throughout radiology, including adbominal, cardiac, and lung imaging.^22^

For an introduction to additional applications not covered here, we recommend Campbell-Washburn et al.^22^

## High Acuity Brain Imaging

High acuity brain imaging in critical care or emergency room patients primarily aims to identify acute problems that require immediate intervention, such as stroke, hemorrhage, edema, and mass effect. Importantly, transporting critically ill patients that require life-sustaining equipment and continuous monitoring outside the intensive care unit (ICU) is difficult, time-consuming, and poses risk of adverse events. Stroke is a leading cause of morbidity and mortality worldwide, causing an estimated 6.5 million deaths each year.^60^ The main stroke types are ischemic and hemorrhagic, with ischemic strokes accounting for 87% of United States cases.^61^ Quickly differentiating between ischemic and hemorrhagic strokes is an essential first step toward treatment. The irreversible infarct core enlarges over time, and evidence supports a 3–4.5-hour treatment window for intravenous thrombolysis and a 24-hour window for mechanical thrombectomy, after which outcomes are considerably worse.^62,63^

Computed tomography (CT) and MRI are the dominant methods for determining stroke subtype.^64^ Provided that MRI is readily accessible, the American Academy of Neurology recommends MRI over CT because it avoids ionizing radiation and has superior soft-tissue contrast, facilitating detection of smaller infarcts.^65,66^ Diffusion-weighted imaging (DWI) has exquisite sensitivity and specificity for ischemia detection, with other sequences, such as fluid-attenuated inversion recovery (FLAIR) and gradient echo, providing complementary information.^65,67^ While MRI is diagnostically superior to CT, conventional MRI is more expensive, not always readily available, and is contraindicated in 10% of patients (eg patients with foreign metal bodies, device implants, claustrophobia, etc.).^68^

In the 1990s and early 2000s, several studies explored diffusion-weighted and perfusion-weighted sequence development for stroke diagnosis on lower-field scanners (0.1–1.0T range).^69^ These studies employed fixed MRI systems with either permanent magnets (typically <0.35T) or superconducting magnets (typically >0.5T). While these systems could detect strokes, sensitivity was reduced compared to 1.5T systems^70^ or scan times were not clinically feasible.^71^

**FIGURE 3:**
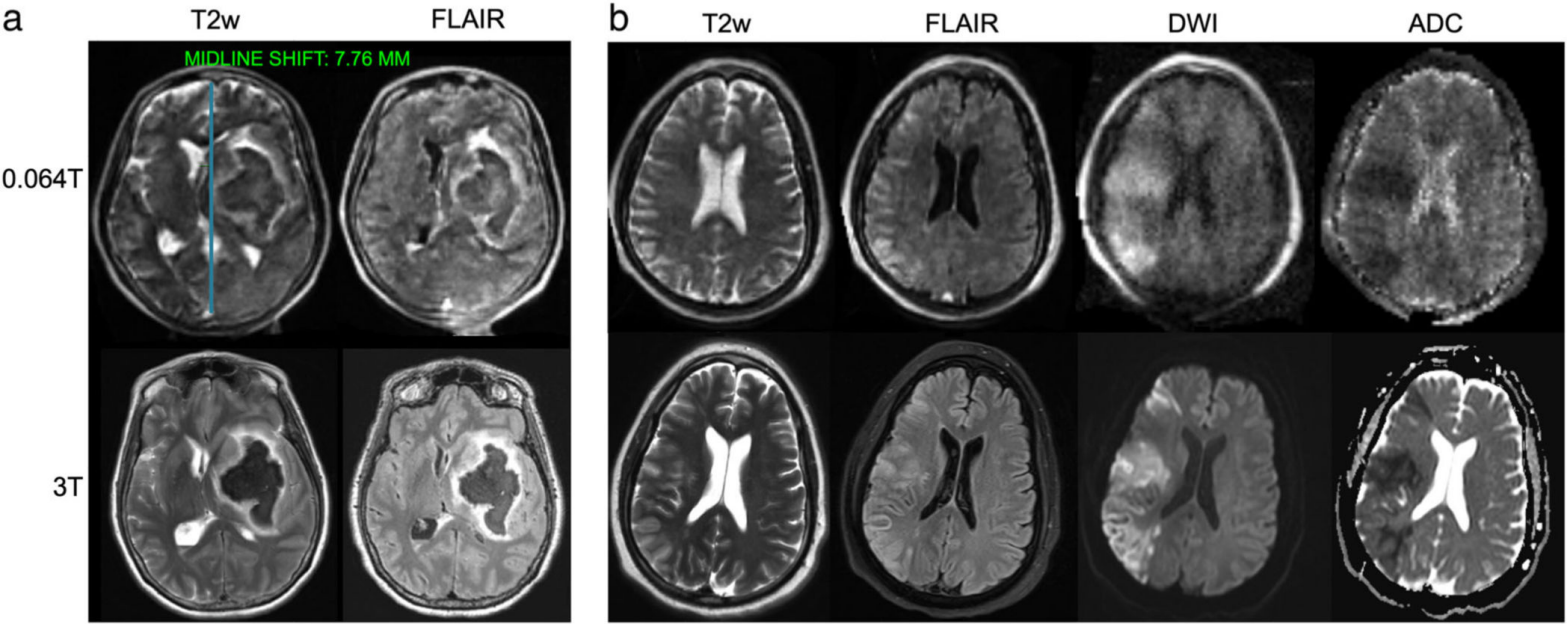
Ischemic and hemorrhagic stroke imaging at 0.064T (top) and 3T (bottom). **(a)** A patient with left basal ganglia intraparenchymal hemorrhage with intravascular extension and midline shift. Midline shift was assessed using an AI-based method embedded in the scanners picture archiving and communication system. The blue line in the top left image indicates the midline shift assessment. **(b)** A patient with right middle cerebral artery acute ischemic stroke image. Images provided courtesy of Dr. Kevin Sheth and Mercy Mazurek, Yale University.

At very-low-field strengths (<0.1T), there have been several recent clinical developments related to stroke imaging. The first report of portable MRI for stroke was published by Sheth et al in 2020.^17,72^ The authors used a 0.064T system to image 30 ICU patients with known intracranial abnormalities, including ischemic stroke, hemorrhagic stroke, subarachnoid hemorrhage, traumatic brain injury, and brain tumor. Bedside imaging was performed with medical equipment being actively used, including ventilators, dialysis machines, and patient monitoring equipment. The portable MRI detected intracranial abnormalities in 97% (28/29) of patients with SOC imaging findings, with one diffuse subarachnoid hemorrhage case missed. In a follow up study, researchers evaluated 144 portable MRI examinations with intracerebral hemorrhage (ICH, N = 56), non-hemorrhagic acute ischemic stroke (AIS, N = 48), or healthy controls (N = 40).^73^ ICH classification accuracy was 90% (130/144), with 80% (45/56) sensitivity. AIS and healthy controls were correctly identified as parenchymal hemorrhage free in 97% (85/88) of cases. Manual hematoma segmentation volumes were strongly correlated between the 0.064T system and SOC imaging. Hematoma volume also correlated with cognitive status (*ρ* = 0.75/0.8, *P* < 0.001) and functional outcome at discharge (*ρ* = 0.59/0.64, *P* < 0.001). Figure 3 provides examples of ICH and AIS at 0.064T compared to 3T imaging. He et al also reported development of a 0.05T scanner with T1 and T2 weighted imaging in three stroke patients.^17^ The authors illustrated ischemic and hemorrhagic stroke cases and longitudinal monitoring of a hemorrhagic stroke with eight scans over 17 days.

The 0.064T system received FDA clearance early in the COVID-19 pandemic.^74^ In their initial publication, Sheth et al imaged an additional 20 ICU patients diagnosed with COVID-19 that presented with altered mental status.^72^ Abnormal findings were present in 40% (8/20) of patients. Turpin et al also described the use of portable MRI in ICU patients with COVID-19, with abnormal findings present in 63% (12/19) of patients.^75^ The researchers highlighted that in five cases portable MRI led to changes in patient management. Importantly, portable MRI can aid in infection control by providing medical imaging to patients inside isolation wards, limiting infectious patient transport.

Additional studies characterized midline shift (MLS) in stroke patients on the 0.064T system.^76,77^ In a 102 patient cohort, low-field MRI had 93% sensitivity and 96% specificity for detecting MLS presence when compared to SOC imaging based on manual identification of midline structures.^76^ In a follow-up study, the commercial AI-based method for assessing MLS (Fig. 3a), which is available at the point-of-care, was non-inferior to neuroradiologists (*P* < 1e-5).^77^ Automated, quantitative biomarkers in lower-field, point-of-care imaging have potential to facilitate interpretation and may extend services to sites where radiologists are not readily available, provided findings are actionable in that context.

In stroke cases time is brain.^78^ Mobile stroke units were developed in 2015 to deliver appropriate therapy as quickly as possible.^79^ These vehicles are equipped with point-of-care lab testing, a CT scanner, and personnel trained in stroke therapy. A recent study with over 1000 patients found mobile stroke units with onboard CT improved patient disability outcomes, reduced time from stroke onset to tissue plasminogen activator administration by 34%, and decreased mortality rate from 11.9% to 8.9%.^80^ While MRI has not previously been integrated into mobile stroke units because of siting and cost issues, new portable low-field MRI systems have been placed in vehicles for remote imaging.^42,43^ Mobile stroke units combined with portable MRI could offer rapid stroke imaging with the high tissue contrast and safety benefits of MRI.

Image quality for some sequences at very-low-field are currently below clinical expectations. In a recent study, two neurosurgeons and a neuroradiologist rated image quality of a portable MRI system using a 5-point scale, 5 being the lowest quality. FLAIR (2.19 ± 0.98), T1-weighted (T1w) (2.6 ± 0.98), T2-weighted (T2w)-axial (2.47 ± 0.99), and T2w-coronal (2.88 ± 0.99) sequences had similar quality ratings with approximately 85% of images deemed adequate for interpretation. However, only 27% of DWI were sufficient for interpretation and images had a correspondingly lower average quality rating (4.13 ± 1.02).^81^ This highlights that DWI, which is the gold-standard for stroke imaging, remains challenging at very-low-field, as this sequence must be fast to avoid motion effects and requires strong gradients with rapid shifting. However, researchers noted DWI quality improved as newer software and hardware versions were released throughout data collection.^73^ In addition, Mazurek et al illustrated several false-negative cases where pathology could be seen upon closer examination, indicating neuroradiologists may require training on lower-field images to become accustomed to their appearance.^73^ Protocols have been described for integrating portable, very-low-field devices into ICU, emergency department (ED), and COVID-19 care settings.^75,81^

**FIGURE 4:**
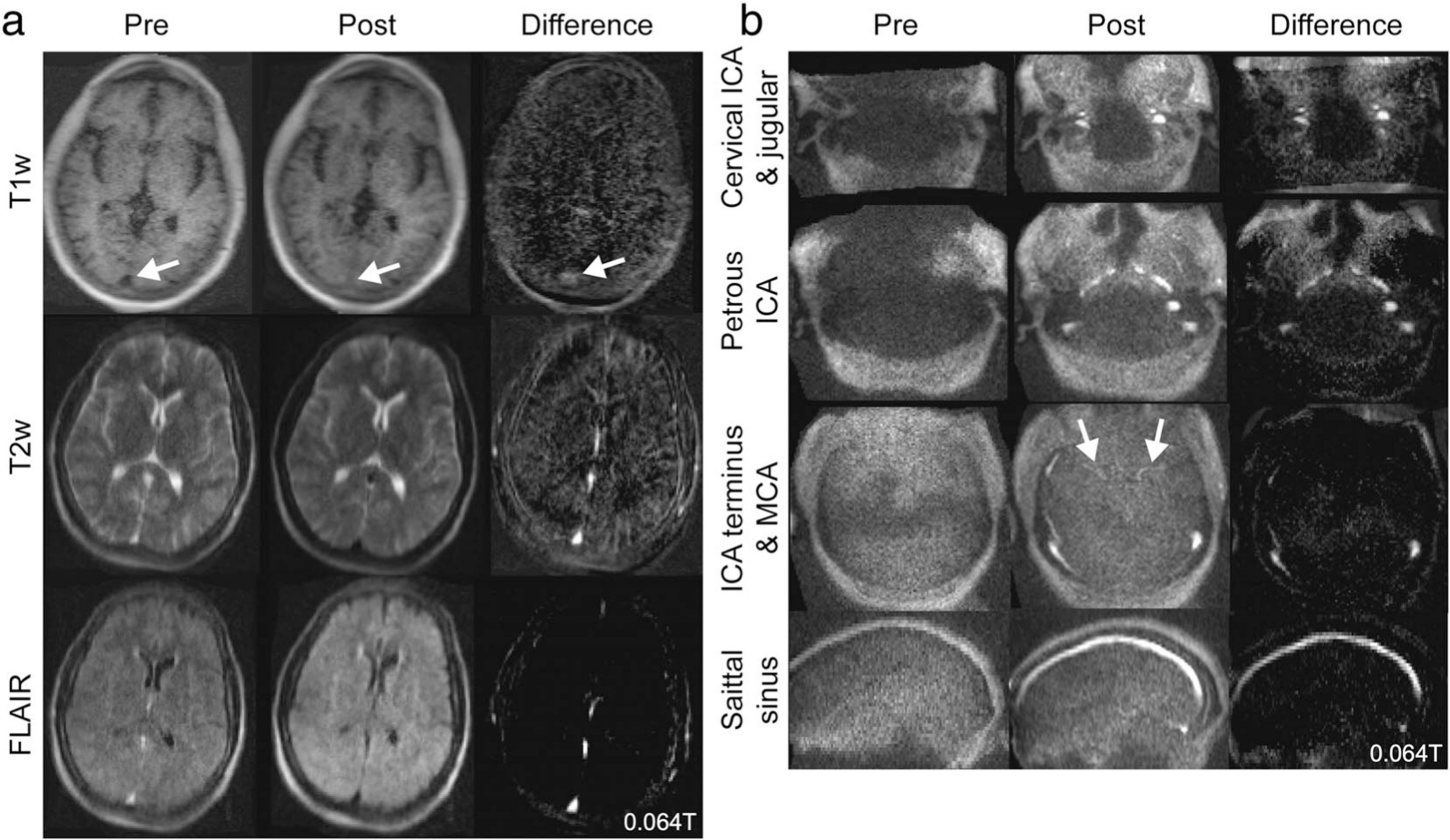
High relaxivity contrast at 0.064T. **(a)** Images from a 27-year-old patient treated with ferumoxytol for iron deficiency anemia. Ferumoxytol is a high-relaxivity contrast agent, which causes venous structures to be hyperintense on T1 and hypointense on T2 and T2-fluid-attenuated inversion recovery. **(b)** The same patient was imaged with a magnetic resonance angiography sequence to visualize arterial structures, including the internal carotid artery (ICA) and middle cerebral artery (MCA).

At stroke centers, vascular imaging also guides acute and chronic management. Both CT angiography and high-field time-of-flight and contrast-enhanced MR angiography exquisitely depict cerebrovascular anatomy. Facilitated by artificial intelligence based processing, CT perfusion determination of infarct core, penumbra, and collateral flow has also become integrated into decision-making for mechanical thrombectomy.^82^ At very-low-field strengths, vascular and perfusion imaging will be challenging and gadolinium contrast will be less effective. Alternative agents, such as superparamagnetic iron oxide nanoparticles, demonstrate high sensitivity even at ultra-low-field strengths (<0.01T).^83^ These agents have been used for vascular imaging at higher field strength, though clinical translation is limited by off-label use and cumbersome administration requirements.^84–86^ In preliminary studies we investigated vascular imaging using ferumoxytol, an iron oxide agent that is FDA approved for treatment of iron deficiency anemia. With high T1 and T2/T2* relaxivity and prolonged intravascular time, this agent represents a best-case scenario for vascular enhancement. Anemia patients were imaged on the portable 0.064T scanner before and after ferumoxytol administration. Visual enhancement of dural venous sinuses and large arteries was observed (Fig. 4), although additional studies of dose and sequence optimization are needed.^87^

To date, ICU stroke imaging is the most well studied clinical application for the new generation of very-low-field devices. While there are technical challenges that must be overcome (Table 2), the technology has been integrated into clinical workflows and demonstrated high stroke sensitivity. Although diffusion sequences on earlier low-field scanners had reduced sensitivity and longer scan times, newer lower-field machines are equipped with state-of-the-art gradient systems, which could improve DWI and increase utility for stroke imaging.^21,22,88,89^ In high resource settings, ED and ICU care are areas where we are likely to see lower-field MRI integrated into SOC practices because of lower device costs, increased device portability, and MR compatibility. First, lower-field scanners are often less expensive to purchase and operate. Several research groups have reported developing systems for under 20,000 USD and commercial systems are available for ~75,000 USD/year.^16,36,37^ Portability of lower-field systems can also increase availability. Portable CT scanners provide an alternative in some centers, but portable MRI could still offer lower cost, more definitive imaging for stroke, with potential for frequent follow-up imaging without ionizing radiation. Finally, many patients with conventional MRI contraindications (eg pacemakers, defibrillators, implants, foreign metal bodies) can be safely imaged on some lower-field systems.^90^ Given that stroke patients may be incapacitated during imaging, reduced screening requirements could be a substantial benefit. For more details on stroke imaging on lower-field MRI the authors recommend Bhat et al.^67^

## Outpatient Neuroimaging

While portable MRI applications have centered on the neuro ICU, evidence is emerging regarding their efficacy for outpatient neurology use cases. Neurological disorders affect 1 billion people worldwide.^91^ They are the leading cause of disability and the second leading cause of death, killing an estimated 9 million people annually.^92^ Many neurological disorders require frequent imaging and longitudinal monitoring. In this section, we review emerging clinical evidence for lower-field devices in outpatient settings and discuss how reduced costs and increased portability could impact when and where patients receive neuroimaging.

**FIGURE 5:**
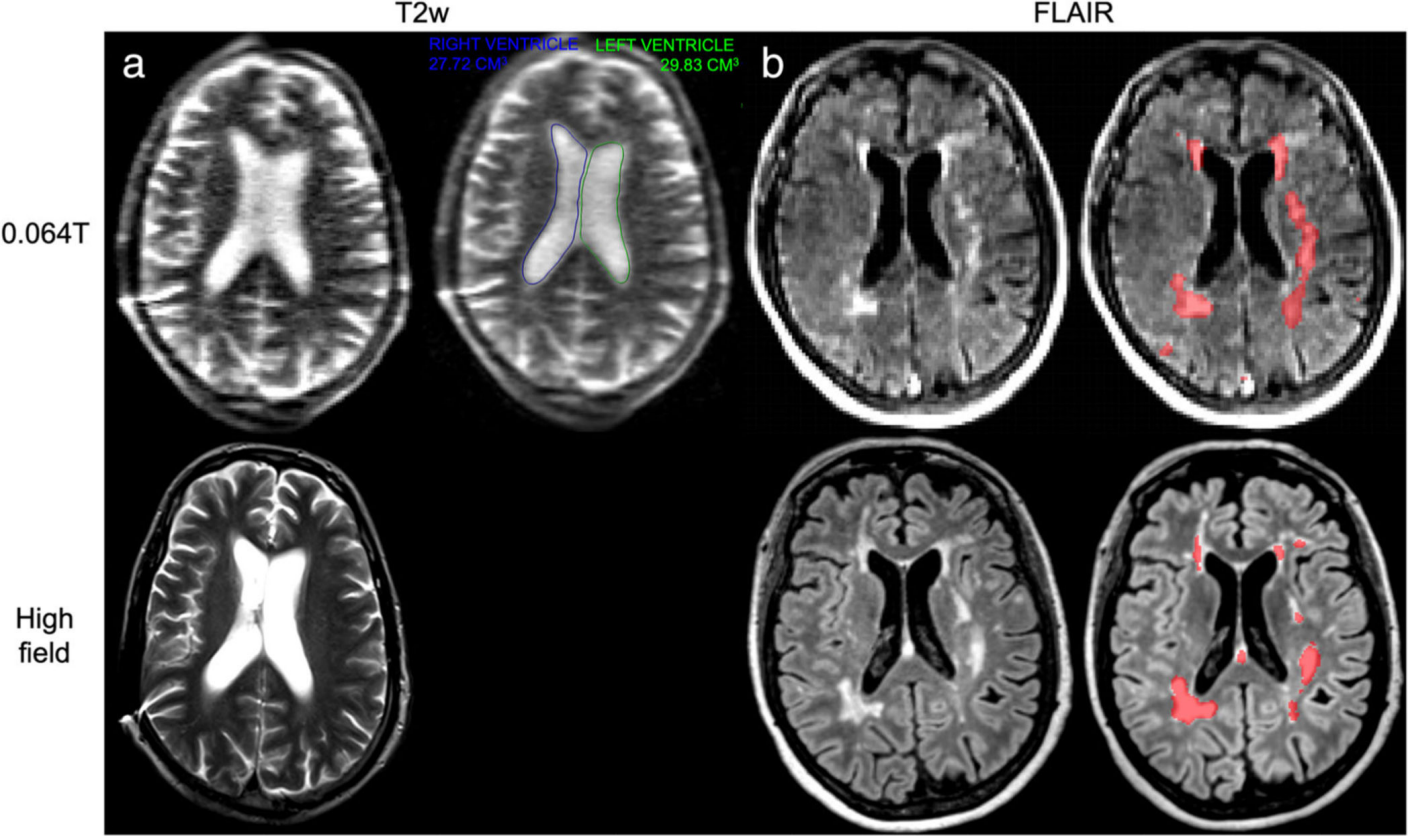
Hydrocephalus and multiple sclerosis patients imaged at 0.064T (top) and high-field (bottom). **(a)** T2w scans of a 55-year-old male with a history of hydrocephalus imaged at 0.064T (scan time: 7:01 minutes) and high-field. A deep learning-based ventricular segmentation algorithm was applied to 0.064T imaging to determine ventricular volumes. Data and algorithm output were visualized in the scanner’s picture archiving and communication system. The right ventricle (blue line) had a volume of 27.72 cm^3^, while the left ventricle (green line) had a volume of 29.83 cm^3^. **(b)** Fluid-attenuated inversion recovery imaging of a 66-year-old female with relapsing–remitting multiple sclerosis at 0.064T (scan time: 9:29 minutes) and 3T (scan time: 5:02 minutes). Automated segmentations of lesions generated using the respective images are overlaid in red on the righthand side.

Hydrocephalus is a condition in which the brain’s fluid-filled ventricles become abnormally enlarged. It is readily managed by placing a sunt to relieve the abnormal accumulation of cerebrospinal fluid but requires imaging for diagnosis and to monitor for under or over shunting. MRI easily depicts the ventricles and offers an ionizing radiation free alternative to serial CT, particularly for children. Researchers have desinged ultra-low-field scanners for the treatment and monitoring of hydrocephalus, though scanning has been limited to phantoms thus far.^93^ At our center, we collected paired SOC and portable very-low-field data from 22 adult hydrocephalus patients and compared ventricle volume estimates to high-field imaging (Fig. 5a).^94^ Radiologists were able to accurately categorize patient ventricle sizes as small, large, mixed, or within normal limits. Ventricle volumes measured at 0.064T and high-field were strongly correlated. Figure 5a illustrates an example of automated ventricular segmentation provided by the point-of-care scanner. In many patients, neurosurgeons implant programmable shunts so fluid drainage can be adjusted non-invasively. Programmable shunts use a magnetic mechanism for drainage adjustment that can be reset by high-field MRI and must be checked by the neurosurgical team after scanning. Lower field strength devices might have reduced interference with shunt settings, but we found that shunt settings were altered by the very-low-field MRI.

**FIGURE 6:**
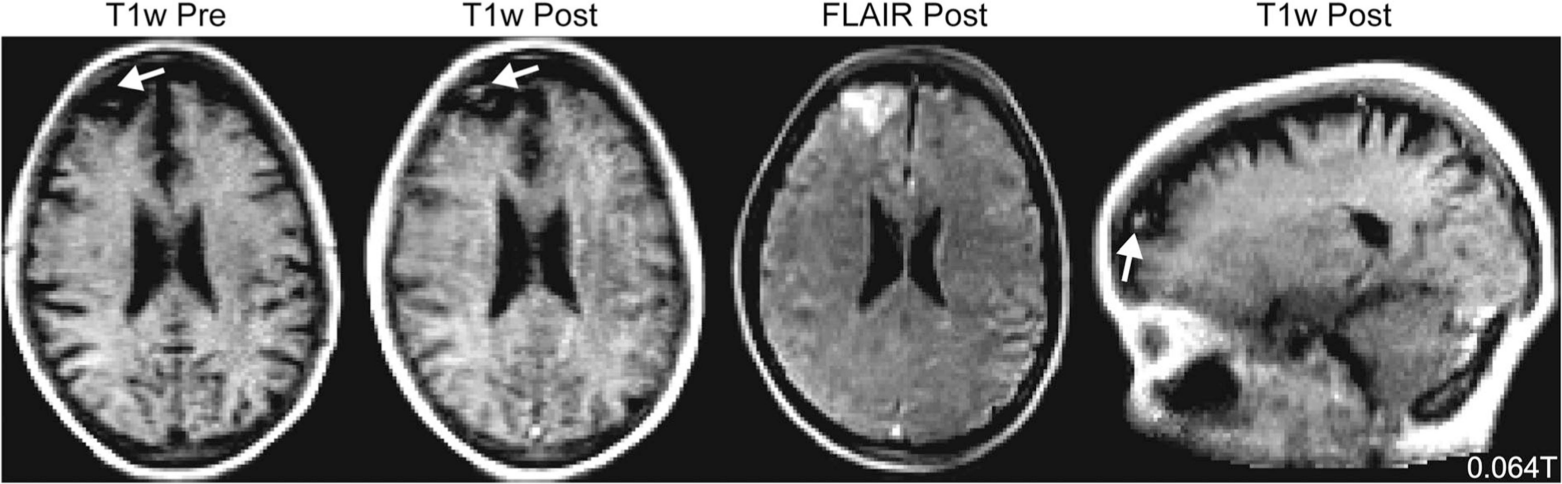
Gadolinium contrast 0.064T. Pre and post gadolinium contrast T1w (scan time: 4:52 minutes) and fluid-attenuated inversion recovery (scan time: 9:29 minutes) imaging in a 54-year-old patient showing an enhancing treated metastasis with surrounding edema. The patient had a history of small-cell lung cancer treated with gamma knife radiosurgery.

Point-of-care scanners have also been used to study multiple sclerosis (MS), a demyelinating disease affecting the brain and spinal cord. Mateen et al used a 0.08T portable scanner to visualize demyelinating lesions in two MS patients.^95^ They noted cortical atrophy in one patient with advanced disease, a finding that has applicability in other neurodegenerative diseases, such as dementia. In a cohort of 36 MS patients, our group probed the sensitivity of a very-low-field device using paired 3T and 0.064T data.^96^ We found the portable 0.064T device was sensitive to white matter (WM) lesions, with both radiologists and automated algorithms able to detect lesions (Fig. 5b). Lower resolution for a given scan time and uncertain benefits from conventional gadolinium contrast are challenges facing very-low-field MRI for MS and other applications that track lesions over time (eg metastases). Initial reports have begun exploring contrast enhancement on very-low-field devices (Fig. 6), but larger studies are needed to optimize sequences and contrast dosage.

In recent decades, researchers have identified MRI biomarkers for neurodegenerative and psychiatric disorders.^97,98^ Disease-specific patterns of brain atrophy can be evident on imaging prior to symptom onset, such as hippocampal volume loss in Alzheimer’s disease.^99^ The presence of other features, such as WM hyperintensities, can further aid in the differential diagnosis.^100^ Imaging biomarkers can also serve as endpoints for clinical trials.^101^ Reduced image resolution in lower-field devices has proven problematic for some conventional neuroimaging pipelines, which could impact biomarker analysis.^102^ To address this, Iglesias et al developed a super-resolution algorithm (SynthSR) that takes lower resolution images and synthesizes a 1 mm isotropic T1w MPRAGE to use for subsequent postprocessing. The group has demonstrated high correlation for key brain regions (eg hippocampus, thalamus, ventricles, cortical gray matter [GM]) between 3T and SynthSR-enhanced lower-field images.^27,103^ Deoni et al demonstrated the ability to generate 1.5 mm isotropic T2w images by registering and averaging three orthogonal slice plane acquisitions.^26^ Although high-field biomarkers have yet to be validated on very-low-field imaging, these initial super-resolution results provide a promising avenue.

In summary, although lower-field devices are not indicated for all applications, growing literature supports the use of in specific cases, such as hydrocephalus and MS. Potential use cases including longitudinal volume tracking (eg hydrocephalus, subdural hematoma) and more frequent MS follow up. Rapid advances in this lower-field technology, aided by machine learning, may expand clinical applications over time (Table 3).

## Intraoperative MRI and MRI-Guided Procedures

MRI is an integral part of neurosurgery, allowing surgeons to plan procedures and monitor for complications. Accurate localization of structures is perhaps the most important problem at the interface of neuroimaging and surgery. In the 1980s, frame-based stereotaxy became the first widely used systematic method for localizing intracranial structures.^104^ These systems fix the patient’s head in a physical frame to relate a coordinate system and have largely been replaced by frameless neuronavigational systems, which rely on fiducial markers.^105^ Today, frameless neuronavigational systems are the most widely-deployed localization method used in HICs.^106^

Despite their physical differences, both frame-based and frameless methods for neuronavigation rely on preoperative imaging. However, significant anatomical distortions occur as tissue is removed and cerebrospinal fluid (CSF) is lost during surgery and neither method permits intraoperative monitoring for complications, such as hemorrhage. In the late 1990s, researchers began developing intraoperative MRI (iMRI) approaches using low-field scanners. Two initial iMRI approaches were developed: the Boston concept,^107^ where surgery is performed in the scanner, and the Heidelberg concept,^108^ where the patient is transported to a nearby scanner. Later, the idea of bringing the scanner to the patient was explored using both high-field^109^ and low-field^110^ devices. Approaches where the patient or the scanner are moved to acquire imaging have become more widely adopted because they permit higher field-strength magnets, unrestricted patient access, and traditional ferromagnetic surgical instruments.^111^

iMRI has largely been pioneered in neurosurgery for brain tumor resections,^112–114^ where the superior soft tissue contrast and 3D visualization of MRI facilitates maximal tumor resection, minimal healthy tissue removal, and monitoring for surgical complications. Other common uses for iMRI include accounting for brain shift during surgery,^115^ biopsy needle guidance,^116^ functional MR guidance to avoid eloquent cortex,^117^ tractography to avoid major WM tracts,^118^ thermal ablation guidance and temperature monitoring,^119^ seizure focus resection,^120^ and intracranial device placement.^121^ Several innovative approaches in mid-to-low-field iMRI have been developed, including the original 0.5T Signa SP (GE) pioneered by Black et al,^107^ the 0.2T Magnetom Open (Siemens) pioneered by Tronnier et al where patients were moved intraoperatively,^108^ and the semi-portable 0.12–0.3T PoleStar N-10, N-20, and N-30 systems (Odin Medical, later Medtronic).^110^ While studies using mid-to-low-field devices demonstrated improvements over standard surgery, including improvements in gross total resection,^122^ remission rate,^123,124^ survival time,^112,113^ there have also been reports of tumor remnants found postoperatively using high-field imaging.^124–126^

While iMRI has explored a range of field strengths, today most devices are 1.5T or 3T. Higher-field strengths are favored because they permit higher image quality with faster acquisition times,^127^ allow for a greater resection extent,^128^ and have increased sensitivity to enhancing neoplasm,^127,129^ which is a primary predictor of surgical outcome. High-field systems have demonstrated clinical and economic benefits,^130^ while evidence for low-field systems has been mixed.^131^ However, high-field devices have disadvantages associated with their increased magnetic field strength, including increased susceptibility artifact, hardware interactions, shielding requirements, a larger 5-gauss line, increased safety precautions, and MR compatibility issues (eg device heating, projectile risk, and image artifacts).^127^ Additionally, iMRI systems require significant capital investment,^132^ more staff training,^132^ and longer procedure times,^111^ which has likely slowed adoption.

**FIGURE 7:**
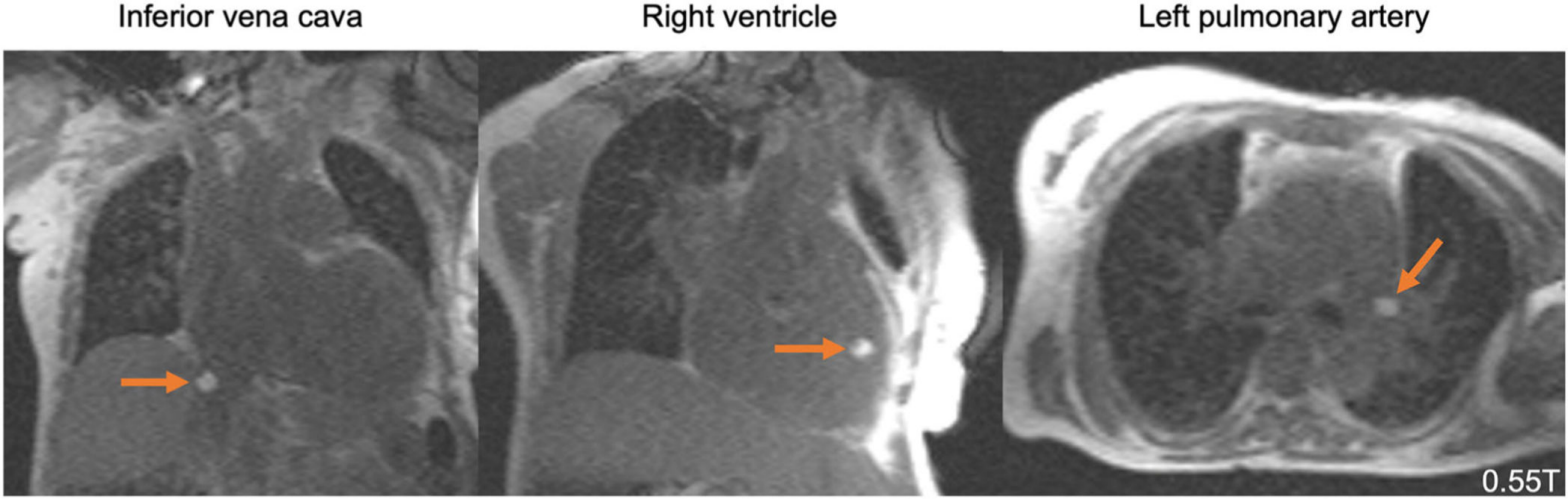
MRI-guided right heart catheterization at 0.55T. Due to device heating, this nitinol guidewire with a gadolinium filled balloon tip catheter could not be used in a 1.5T scanner, but can be safely used for 0.55T MRI-guided procedures. The gadolinium filled balloon is used to navigate the guidewire during right heart catheterization. Images acquisitions are real-time bSSFP with partial saturation pre-pulse (TE = 2 msec, TR = 4 msec, flip angle = 45°, partial saturation pre-pulse flip angle = 60°). Images provided courtesy of Dr. Adrienne Campbell-Washburn, National Heart, Lung, and Blood Institute.

Newer lower-field devices may overcome some disadvantages of high-field scanners while improving upon shortcomings from prior lower-field iterations. With a sufficiently low magnetic field, surgeries can be performed using traditional implements without moving the patient or scanner. Additionally, staff training burden and safety precautions are reduced. Some researchers have advocated for the development of mid-field systems equipped with the latest technology developed for high-field systems.^21,22,88,89^ Campbell-Washburn et al modified a 1.5T Siemens Magnetom Aera to operate at 0.55T while maintaining gradient performance and using a 16-channel head coil.^22^ A similar design is now commercially available as the 0.55T Siemens Magnetom Free.Max. Campbell-Washburn et al described seven patients that underwent successful MRI-guided right heart catheterization using the mid-field scanner and demonstrated reduced RF-induced heating in guidewires, catheters, and pacemakers previously deemed unsafe at 1.5T (Fig. 7).^22,133^ Synaptive Medical offers the 0.5T Evry system, which was designed for iMRI applications. Preliminary studies report reduced risk of RF-induced heating and have demonstrated gradient specifications (max amplitude = 100 mT/m, max slew = 400 T/m/sec) that enable high-quality diffusion tensor imaging.^134–136^ Additionally, MRIdian’s 0.35T ViewRay system for MRI-guided linac radiation therapy received FDA-clearance in 2014.^137^ This device combines low-field MRI with an MR compatible radiation therapy system to permit precise tumor localization and monitoring during treatment. To our knowledge, no publications have reported new mid-field scanners being used for peri-neurosurgical or neurointerventional procedures to date, but their increased surgical precision and MR compatibility alongside the potential for reduced iMRI costs merit further investigation.

At very-low-field strengths, the 0.066T Promaxo system has been proposed as a point-of-care method for guiding prostate biopsies by urologists.^138,139^ Prior to the procedure, high-field imaging is collected to identify biopsy targets. Very-low-field imaging is collected during the procedure and registered to high-field imaging to provide needle guidance. In phantom studies, needle guidance error was less than 3 mm on average. In early in-vivo reports, MRI guidance increased sensitivity to prostate cancer by 37% over blinded systematic biopsy (MRI-guided: 12/16, blinded systematic biopsy: 6/16). To date, there are no reports of new lower-field systems for neurosurgical applications. However, prior iterations, such as the discontinued PoleStar system, had operational cost of approximately 750,000 USD annually.^131^ New lower-cost scanners have potential to change the costbenefit analysis and may drive iMRI adoption in areas previously deemed cost-prohibitive.^131^

Although new low-to-mid field devices offer reduced scanner costs and have improved in image quality, they still face significant challenges (Table 4). Device sensitivity is the most critical question. Prior studies of low-field devices frequently reported tumor remnants on high-field follow-up. Gadolinium contrast is proportional to magnetic field strength, which is another concern. While conventional contrast usage may be adequate for mid-field scanners, to achieve sufficient contrast at even lower field strengths we may need to increase dosages or develop higher relaxivity agents.^84^ Finally, magnet and coil configurations must be designed to accommodate surgical implements, monitoring equipment, and staff needs during surgery. For more details on iMRI applications, the authors recommend Hall et al.^121^

## Pediatrics

Pediatric MR imaging is increasingly used for clinical and research purposes given concerns of ionizing radiation exposure from CT and x-ray.^140^ There are unique challenges associated with imaging pediatric patients, including safety concerns, image acquisition obstacles, and differences in image analysis.^141^ These barriers can prevent direct translation of research or clinical practices from adult to pediatric populations and highlight the importance of evaluating each step of neuroimaging pipelines with the target population in mind. Here, we highlight some features of pediatric neuroimaging and discuss lower-field MRI contributions.

Ultrasound (US), CT, and MRI are the dominant modalities for pediatric neuroimaging. US is primarily used to diagnose disorders such as hydrocephalus and intracranial hemorrhage in the first 6 months of life, prior to anterior fontanelle closure.^142^ CT is commonly used for trauma-related neuroimaging because it offers fast imaging with good contrast between blood, bone, and brain.^142^ CT acquisition times are short so sedation of pediatric patients is rarely required. However, CT has less soft tissue contrast and exposes patients to ionizing radiation, making it less desirable for repeated imaging, such as monitoring shunted hydrocephalus patients.^143,144^ While MRI offers superior soft tissue contrast and non-ionizing radiation, it is more expensive and less widely available than CT. This is particularly relevant in rural areas and in many LMICs.^145^ MRI acquisition times are longer than CT, often necessitating patient sedation which poses safety risks.^141^ Finally, MRI hardware, such as head coils, needs to be optimized for pediatric patients.^146^

Open scanner designs and reduced scanner noise are appealing advantages of lower-field systems. Patient compliance can be particularly challenging for children under 6 years old, who often require sedation.^141^ With open scanner designs, claustrophobia is less of a problem. Sedation usage may also decrease, as parents or providers can access children during scanning. Rupprecht et al compared sedation and anesthesia requirements of 274 pediatric patients imaged on a 0.2 tesla open bore MRI (Magnetom Open, Siemens) to 111 patients imaged on a standard high-field, closed bore system.^147^ In the open system, only 27% (74/274) of patients required sedation compared to 47% (52/111) on the closed system. The effect was most pronounced in children under 10 years old. The authors also reported using lower sedation doses and that monitoring patients was easier. Moreover, lower-field devices have reduced acoustic noise, which is particularly useful for imaging infants, who are often asleep during scans or require ear protection.^39,141^

**FIGURE 8:**
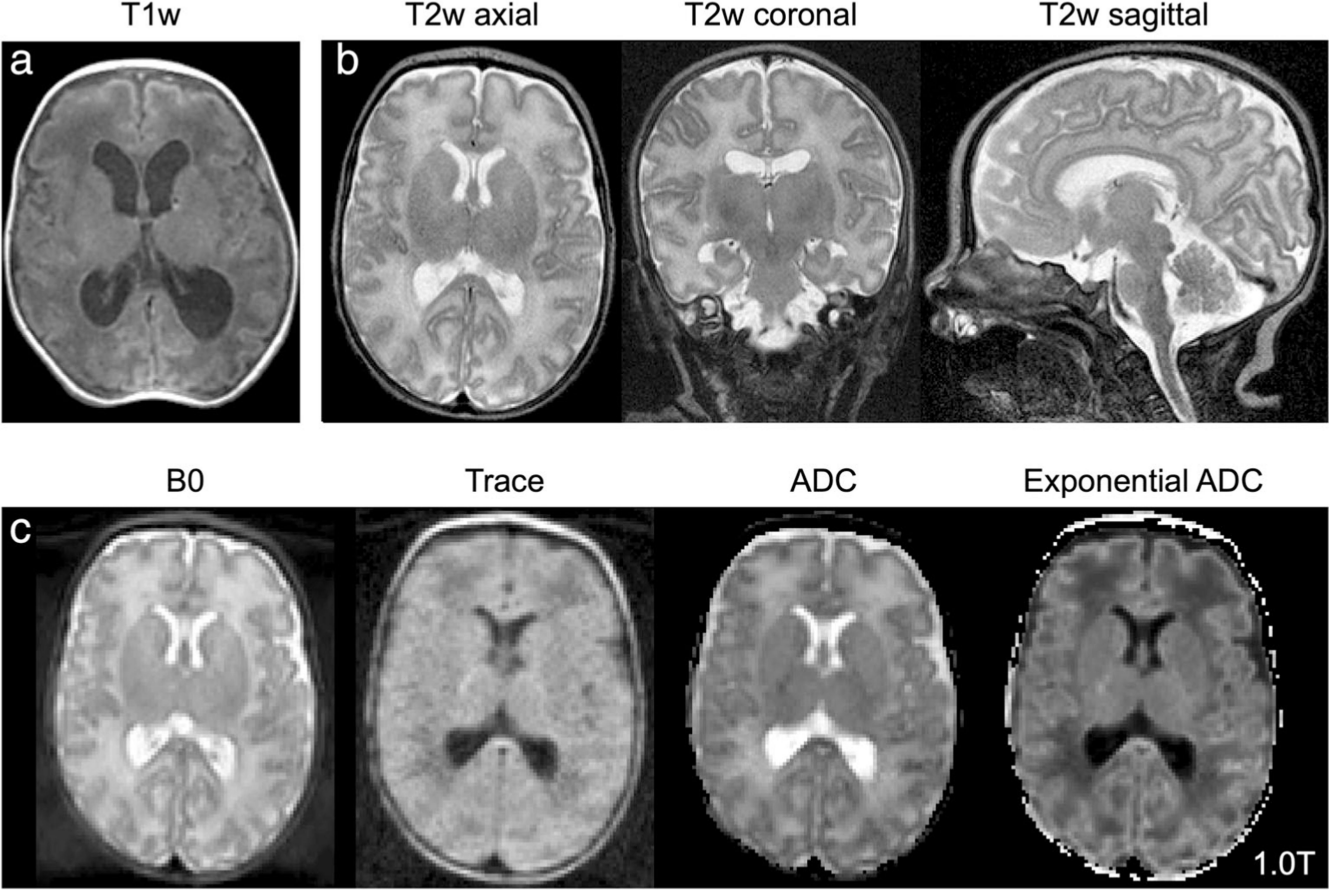
Images from pediatric patient imaged at 1T. **(a)** T1-weighted, **(b)** T2-weighted (left to right: axial, sagittal, coronal acquisitions), and **(c)** diffusion imaging (left to right: B0, trace, apparent diffusion coefficient [ADC], exponential ADC). Images provided courtesy of Wendy Slatery and John Posh, Aspect Imaging.

Lower-field systems have been deployed for thoracic, orthopedic, and neurosurgical applications^148,149^ and have been integrated into pediatric and neonatal ICU settings to reduce patient transport risk.^150,151^ In the early 2000s, Whitby et al compared SOC imaging (US) to a 0.17T (InnerVision MRI Ltd, London, UK) in 134 neonatal patients (89 controls & 43 with suspected pathology).^150^ In 56% (24/43) of patients, MRI provided additional clinical information beyond US findings. In 40% (17/43) of cases, the US read was normal, while MRI detected five subdural hematomas cases, five hypoxic ischemia cases, and seven additional findings. Whitby et al noted device cost (~£150,000 in the United Kingdom) and relative cost per treatment (~£60) were similar between US and MRI.^150^ More recently, Aspect Imaging received FDA clearance for their 1T Embrace neonatal MRI system, which is designed to be embedded in the NICU. Thiim et al compared the 1T system to US in the NICU and conventional 3T scanning with patient transport outside the NICU.^151^ Compared to US, the 1T scanner provided significant clinical benefit, with abnormal findings identified in 15 additional cases (1T MRI: 59, US: 44). The authors reported greater sensitivity to WM injury (1T MRI: 17, US: 7), hypoxic ischemia (1T MRI: 2, US: 0), and hemorrhage (1T MRI: 25, US: 20). For 32 patients, 3T comparison imaging was collected. Reports of brain injury were largely concordant between 3T and 1T, with two notable exceptions: one case of punctate susceptibility foci noted at 3T but not 1T and one polymicrogyria case noted at 1T but not 3T. Figure 8 illustrates example images of neonatal patients on the 1T scanner.

Portable very-low-field systems have also been applied to pediatric neuroimaging. Deoni et al described the first use of a portable 0.064T MRI to track neurodevelopment in a cohort of 42 healthy children (age range: 6 weeks to 16 years).^102^ The researchers calculated brain volume estimates for GM, WM, and CSF and replicated known developmental trajectories from 3T imaging studies. The authors reported greater scan success rates at 0.064T (89%) than 3T (75%), presumably related to the open bore design. However, researchers noted that some sequences and analytical software may require optimization to work in pediatric populations. The team’s volumetric analyses were performed on T2w sequences, which offered superior anatomical contrast, instead of the T1w sequences typically used in morphometrics. WM myelination changes rapidly during childhood, and developing brains have higher water content in unmyelinated WM. Both water content and magnetic field strength influence tissue relaxivity properties.^146,152^ The researchers used sequences designed for adult imaging, which need to be optimized based on pediatric patient age to provide better tissue contrast. Additionally, the researchers utilized two common neuroimaging analysis software packages, Advanced Normalization Tools and FreeSurfer. They noted that FreeSurfer failed to process the low-resolution data accurately, although this may improve with sequence optimization, super-resolution approaches, and further software development. ^28,103^ Finally, the researchers only replicated group-level developmental trajectories, and it remains to be seen if accurate volume estimates can be obtained for individual patients.

The Deoni et al study highlights how expanded access to low-cost scanners could change how large neuroimaging studies are conducted. These devices could enable larger sample sizes by reducing per patient scanning costs and shift recruitment focus away from academic hospitals in HICs to include more children from rural areas and LMICs. A group at Queen Elizabeth Central Hospital in Malawi recently reported scanning 260 patients with a 0.064T portable MRI, including examples of malarial encephalopathy and subdural empyema, a collection of purulent material around the brain.^153^ Research applying low-field MRI in LMICs is likely to increase in coming years with increasing investment in the field.

Despite the many advantages lower-field MRI offers for pediatric neuroimaging, there are significant barriers that must be overcome before the technology can be widely adopted (Table 5). Foremost, sequences, hardware, and analytic software need to be optimized for pediatric populations. Multiple researchers have reported that T1w and DWI sequences require quality improvements and optimization for pediatric patients.^75,81,102^ Many current lower-field systems contain hardware designed for adults, which may require alterations for pediatric patients.^146^ Additionally, on scanners below mid-field strength some sequences used for neurodevelopmental research are difficult to obtain, including functional, perfusion, and high-angle-diffusion sequences. In lower resolution images, it may be more feasible to place individuals on growth trajectories, measure macroscale volumes, and characterize patterns of neurodegeneration rather than to quantify the volume of smaller brain structures. Finally, because of the reduced SNR per unit time at lower magnetic field strengths, sequences are often longer, which reduces patient compliance and exacerbates motion issue

## Musculoskeletal Imaging

Orthopedics and musculoskeletal imaging (MSK) were early adopters of low-field MRI and remain one of the few specialties in HICs where low-field is relatively common in clinical practice today. Low-field scanners are particularly attractive to orthopedics were metal implants are common and unique scanner designs (eg open bores, extremity specific scanners, and vertical scanners for weight-bearing studies) offer more clinically tailored imaging.^154^ Several manufacturers, including FONAR, Esaote, and Paramed currently offer low-field MRI scanners targeted at orthopedic and spinal applications.^155^ These devices focus on minimizing imaging costs or feature rotating tables which permit weight-bearing and kinematic imaging. Unlike conventional closed-bore systems, extremity specific and open bore scanner configurations permit central positioning of limbs in the magnetic field, which increases image quality.^154^ Although these advantages have given low-field scanners some traction in orthopedics, due to their lower resolution they are predominantly used for niche applications.^155^

While low-field MRI offers a cost-effective method for orthopedic imaging,^156^ there remain significant challenges that must be overcome before scanners become more widely adopted. The three primary obstacles are user perceptions of low-field image quality, developing the full range of clinically necessary sequences, and loss of quality control by radiologists. User perception of image quality and its impact on diagnostic value is perhaps the most important problem facing lower-field MRI. In the 1990s and early 2000s, low-field MRI scanners were directly compared to standard high-field systems across a range of orthopedic applications. In shoulder imaging, most studies found equivalent performance between scanners^157,158^ with the notable exception of Magee et al, who reported that subsequent high-field scans changed reviewer interpretations in 9/40 patients.^159^ However, as noted by Thomsen et al, their results were for a single low-field system and may not generalize to low-field devices more broadly.^160^ In knee imaging, most studies reported equivalence,^161^ although a meta-analysis including 29 knee MRI studies identified a significant reduction in diagnostic performance for anterior cruciate ligament (ACL) tears.^162^ Low-field scanners were evaluated for numerous other orthopedic applications, including the elbow,^163^ hand,^164^ and foot.^165^ While many studies reported equivalent diagnostic performance between low-field and high-field scanners, there remained significant concern that the lower SNR per unit time would translate to missed diagnoses and perhaps legal liability.^161^

In the first report describing knee scans on the portable 0.064T scanner, raters visualized the quadriceps tendon, patellar tendon, and posterior cruciate ligament easily, but had more difficulty with the ACL, iliotibial band, medial collateral ligament, and lateral collateral ligament.^166^ Some sequences may need optimization to provide sufficient visualization of key knee anatomy. In the mid-field range, Khodarahmi et al compared artifacts in 0.55T and 1.5T imaging of patients with hip implants. They found a 45%–64% reduction in image artifact when using 0.55T compared to 1.5T, even when 1.5T scanners used a slice encoding for metal artifact correction protocol, though there was a modest 17%–28% reduction in SNR at 0.55T.^167^

**FIGURE 9:**
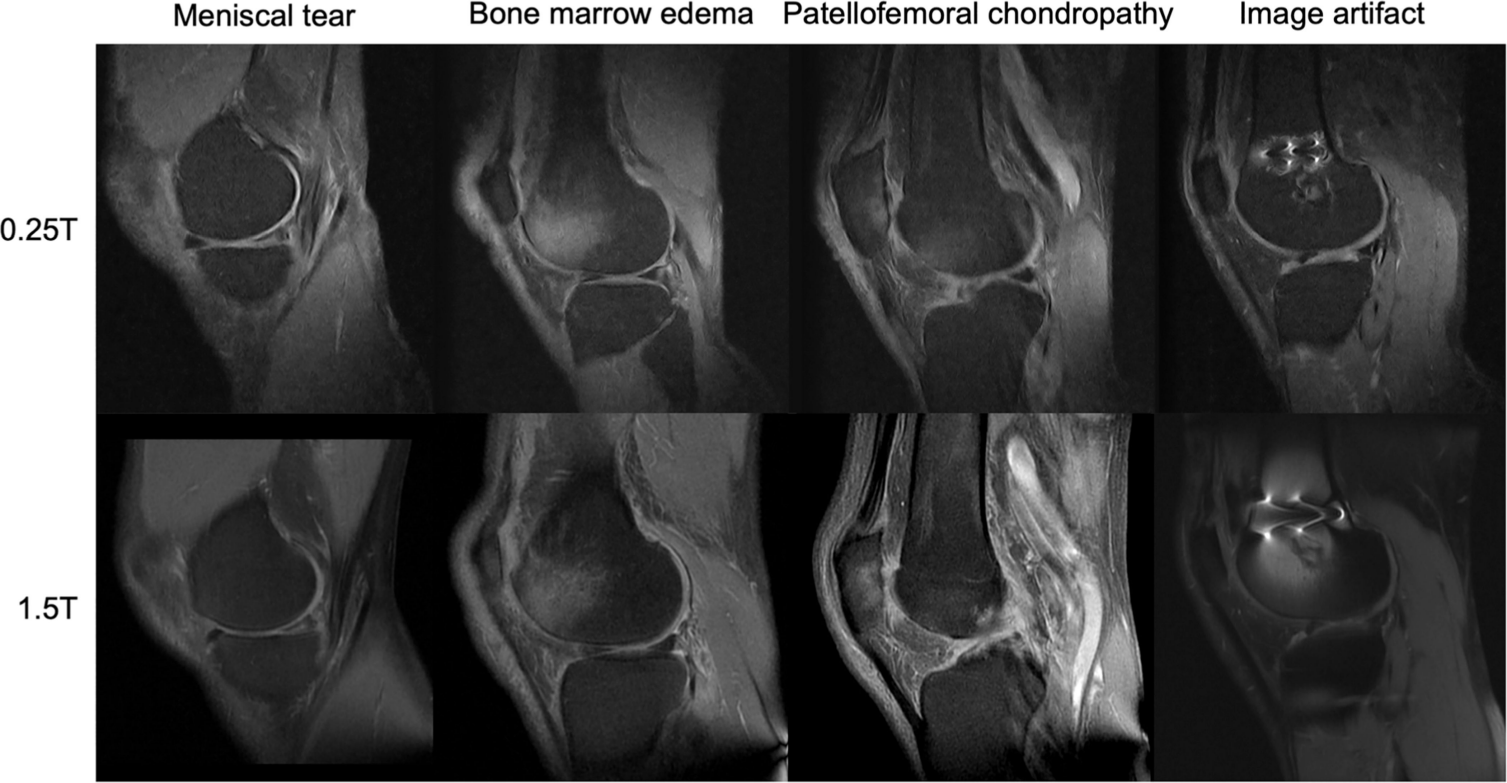
Examples of musculoskeletal imaging at 0.25T (top) and 1.5T (bottom). Images provided courtesy of Dr. Riccardo Monti, Dr. Frederico Bruno, Prof. Antonio Barile, and Prof. Carlo Masciocchi, University of L’Aquila, Italy.

Another challenge at low-field is fat suppression, which increases contrast when evaluating cartilage, menisci, or bone marrow.^168^ Many fat suppression techniques rely on the chemical shift between lipids and water, which is proportional to magnetic field strength.^169^ Bellisari et al demonstrated a fat suppression technique on a 0.25T low-field scanner and found comparable diagnostic performance to 1.5T (Fig. 9).^170^ Although this shows promise, such techniques may not transfer to even lower field strengths and represents only a single sequence among multiple that still need to be replicated and optimized for low-field scanners.

As scanners become increasingly used by non-radiologists and in nontraditional health care settings, there is significant concern that radiologists will lose quality control oversight or be unavailable for image interpretation. Portable, very-low-field devices could be deployed in field hospital or sports arena sidelines for musculoskeletal imaging. Guallart-Naval et al reported the use of a 0.07T device to scan a patient with a knee implant in various nontraditional setting (eg indoors vs. outdoors) and conditions (eg power via wall outlet vs. gas generator).^35^ SNR was most affected in outdoor settings using gas generator power, although even under these conditions SNR was considered acceptable by the authors. Additionally, there was minimal artifact near the participant’s knee implant in all imaging settings. Ownership of imaging quality control may need to be re-evaluated as devices move to nontraditional settings.

The idea of providing diagnostic support to low-field scanners in remote settings has been gaining traction. Raman et al used a neural network to perform binary classification of knee effusion in simulated low-field images.^171^ Their network had comparable accuracy to radiologists (47.2% vs. 41.7%, respectively), indicating that such technology could provide diagnostic feedback when a physician is not readily available. Automated methods for segmenting knee anatomy at low-field also show promise for providing diagnostic support.^172^ While this could further be mitigated by telehealth, it remains unclear how health systems will adapt to increased imaging outside the radiologist’s realm.

Although lower-field devices offer advantages for musculoskeletal imaging and have a demonstrated history in clinical practice, there are significant challenges that the field must still be overcome (Table 6). For more details on low-field orthopedic applications, the authors recommend Ghazinoor et al.^161^

## Conclusion

In conclusion, we have reviewed potential clinical applications for lower-field MRI, discussing how the technology can be deployed to complement existing high-field devices and expand access to imaging where it was previously not economically feasible. When thinking about appropriate clinical applications, opportunities for lower-field strength devices center around their advantages, most notably lower cost and portability. Although this review promotes the clinical translation of lower-field scanners, we must acknowledge that there are outstanding questions surrounding the appropriate implementation of these devices which are scientific, practical, legal, ethical, economic, and cultural. How should new lower-field scanners be integrated with other imaging in highresource settings? How should devices be utilized in low-resource settings, where they may be the only option available? With the advent of portable systems, who will control point-of-care imaging? Can clinicians adjust their expectations around MRI and the level of image quality necessary to answer clinical questions? Can lower-field devices be used for triage or will they simply contribute to more imaging? Finally, for the industry, does economic viability require broader use of lower-field devices in HICs to support dissemination in lower-resource settings? Despite the questions and challenges facing low-field MRI, the technology demonstrates immense clinical promise with potential to increase medical imaging access and improve patient care worldwide.

## Figures and Tables

**TABLE 1.** Advantages and disadvantages of low-field strength MRI relative to standard-of-care high-field MRI

Advantages	Details	Implications
Lower cost	• Cheaper to manufacture, purchase, install, and maintain	• Increased access for clinical care or research
Smaller footprint	• Magnets and other components are smaller and weigh less, no super cooling, less need for dedicated shielded room	• Potential portability • Point of care use
Lower power	• For permanent magnets only electronics and gradients need power, can use regular power outlets, generators, or batteries	• Potential portability • Point of care use
Flexible bore configurations	• C-shaped, wider bore, single-sided, and vertical orientation.	• Decreased claustrophobia • Pediatric imaging • Intraoperative imaging • Tailored scanner designs
Safer	• Less risk of metallic projectiles • Decreased specific absorption rate and device heating • Less risk of device interactions • Decreased acoustic noise	• Potential portability • Point of care use • Intensive care unit integration • Image patients with devices or implants • Pediatric imaging
Relaxivity differences	• Lower specific absorption rate • Shorter T1 • Longer T2/T2*	• Lower device heating and susceptibility artifact • Shorter radiofrequency pulses • Longer spin echo trains
Disadvantages	Details	Mitigation strategies
Lower signal	• Lower SNR per unit time • Decreased resolution • Increased scan time • Decreased field of view • Less benefit from gadolinium	• Signal averaging • SNR-efficient acquisitions • Multiple acquisition planes • Undersampling • Deep learning reconstruction • Selecting appropriate clinical applications
Relaxivity differen ces	• Reduced gray/white contrast • Less chemical shift (eg fast suppression) • Less benefit from gadolinium	• Sequence optimization • Relaxivity efficient sequences • Increased gadolinium dose • Alternative contrast agents • Follow-up high-field imaging

**TABLE 2.** Promise and challenges in high acuity brain imaging

Promise	Challenges
• Low-field MRI can assess stoke with relatively high sensitivity and specificity.	• DWI sequences have lower quality on portable scanners.
• Automated diagnostic tools are being integrated into portable MRI workflow.	• Assessment of stroke penumbra and vasculature remains uncertain.
• Portability could enable mobile stroke units equipped with MRI.	• Reading low-field scans may require retraining for neuroradiology personnel.

**TABLE 3.** Promise and challenges in outpatient neuroimaging

Promise	Challenges
• More frequent scanning for longitudinal follow- up performed at the point- of-care.	• Lower-field strength scanners may still interfere with the function of some implanted devices.
• Reduced scanning expenses could make MRI a cost-effective screening tool.	• Lower resolution and differences in tissue contrast may impact the functionality of existing post-processing software.
• Potential for increased compatibility with implanted medical devices.	• Reduced resolution and gadolinium contrast will likely constrain the range of potential applications.
• Machine learning can enhance image quality & compatibility with post-processing software.	

LMICs = low- and middle-income countries.

**TABLE 4.** Promise and challenges in intraoperative MRI and MRI-guided procedures

Promise	Challenges
• Low-cost systems could alter the economic feasibility of intraoperative MRI guidance for some procedures. • Ability to quickly check for surgical complication, such as hemorrhage, prior to ending the surgical procedure. • Reduced requirements for radiofrequency shielding, operational safety, staff training, 5-gauss line distance, and increased MR compatibility for traditional surgical implements.	• Increased procedure time and device costs may discourage adoption of low-field iMRI devices. • Lower image quality, smaller field-of-views, and reduced scanner versatility. • Reduced gadolinium contrast could affect procedures where resection of enhancing tissue predicts patient outcome. • Temperature resolution decreases with magnetic field strength, affecting real-time MR thermography during ablations. • Device configurations need to be optimized to facility patient access.

**TABLE 5.** Promise and challenges in pediatric and neonatal imaging

Promise	Challenges
• Open bore designs permit patient access for comforting or medical treatment during imaging. • Reduced scanner noise for imaging sleeping infants. • High contrast, non-ionizing radiation method for assessing chronic neurologic conditions, such as hydrocephalus. • Expanded clinical (intensive care and surgical) and research (neurodevelopmental) applications. • Increased imaging access and acquisition of larger neuroimaging studies in LMICs.	• Optimizing sequences (T1w, DWI) for pediatric patients. • Reduced availability of some sequences used in neurodevelopmental research, including functional, perfusion, and high-angle diffusion sequences. • Pediatric sequences are often shorter to combat patient motion; low-field sequences typically have longer acquisition times. • Lower resolution may impact brain volumetric measurements.	

LMICs = low- and middle-income countries.

**TABLE 6.** Promise and challenges in musculoskeletal imaging

Promise	Challenges
• Lower cost permits tailored scanner designs (eg hand, foot, limbs) and integration into orthopedic departments. • Unique scanner designs allow expanded patient positioning and mobility, enabling weight-bearing and kinematic studies. • Open designs permit positioning of limbs in isocenter. • Portable scanners could offer unique opportunities for sports medicine. • Metal artifact from implants and devices is reduced at lower-field strengths.	• Lower resolution and reduced sensitivity to certain anatomic structures. • Less spectral separation between water and fat, making fat-suppression challenging. • Loss of quality control by radiologists.

## References

[1] Hong AS, Levin D, Parker L, Rao VM, Ross-Degnan D, Wharam JF. Trends in diagnostic imaging utilization among medicare and commercially insured adults from 2003 through 2016. Radiology 2020; 294:342–350. 31891320 10.1148/radiol.2019191116 PMC6996668

[2] Geethanath S, Vaughan JT. Accessible magnetic resonance imaging: A review. J Magn Reson Imaging 2019;49:e65–e77. 30637891 10.1002/jmri.26638

[3] Marques JP, Simonis FFJ, Webb AG. Low-field MRI: An MR physics perspective. J Magn Reson Imaging 2019;49:1528–1542. 30637943 10.1002/jmri.26637 PMC6590434

[4] Mollura DJ, Shah N, Mazal J. White paper report of the 2013 RADAID conference: I. J Am Coll Radiol 2014;11:913–919. 25189930 10.1016/j.jacr.2014.03.026

[5] Maru DS-R, Schwarz R, Andrews J, Basu S, Sharma A, Moore C. Turning a blind eye: The mobilization of radiology services in resourcepoor regions. Global Health 2010;6:18. 20946643 10.1186/1744-8603-6-18 PMC2964530

[6] Ginde AA, Foianini A, Renner DM, Valley M, Camargo CA. Availability and quality of computed tomography and magnetic resonance imaging equipment in U.S. emergency departments. Acad Emerg Med 2008;15:780–783. 18783491 10.1111/j.1553-2712.2008.00192.x

[7] Sutliff MH. Contribution of impaired mobility to patient burden in multiple sclerosis. Curr Med Res Opin 2010;26:109–119. 19916707 10.1185/03007990903433528

[8] Sperr E. PubMed by Year [Internet]. 2016 [cited 9/6/2022]. Available from: https://esperr.github.io/pubmed-by-year/

[9] Edelman RR. The history of MR imaging as seen through the pages of radiology. Radiology 2014;273:S181–S200. 25340436 10.1148/radiol.14140706

[10] Moser E, Laistler E, Schmitt F, Kontaxis G. Ultra-high field NMR and MRI—The role of magnet technology to increase sensitivity and specificity. Front Phys 2017;5:33.

[11] Sarracanie M, Salameh N. Low-field MRI: How low can we go? A fresh view on an old debate. Front Phys 2020;8:172.

[12] Sarracanie M, Lapierre CD, Salameh N, Waddington DEJ, Witzel T, Rosen MS. Low-cost high-performance MRI. Sci Rep 2015;5:1–9. 10.1038/srep15177 PMC4606787 26469756

[13] Cooley CZ, McDaniel PC, Stockmann JP, A portable scanner for magnetic resonance imaging of the brain. Nat Biomed Eng 2021;5: 229–239. 33230306 10.1038/s41551-020-00641-5 PMC8597947

[14] O’Reilly T, Teeuwisse WM, Webb AG. Three-dimensional MRI in a homogenous 27 cm diameter bore Halbach array magnet. J Magn Reson 2019;307:106578. 10.1016/j.jmr.2019.106578 31470234

[15] Broche LM, Ross PJ, Davies GR, MacLeod MJ, Lurie DJ. A whole-body fast field-cycling scanner for clinical molecular imaging studies. Sci Rep 2019;9:10402. 31320653 10.1038/s41598-019-46648-0 PMC6639535

[16] Liu Y, Leong ATL, Zhao Y, A low-cost and shielding-free ultralow-field brain MRI scanner. Nat Commun 2021;12:7238. 34907181 10.1038/s41467-021-27317-1 PMC8671508

[17] He Y, He W, Tan L, Use of 2.1 MHz MRI scanner for brain imaging and its preliminary results in stroke. J Magn Reson 2020; 319(106829):106829. 10.1016/j.jmr.2020.106829 32987217

[18] O’Reilly T, Webb A. Deconstructing and reconstructing MRI hardware. J Magn Reson 2019;306:134–138. 31311711 10.1016/j.jmr.2019.07.014

[19] Su J, Pellicer-Guridi R, Edwards T, A CNN based software gradiometer for electromagnetic background noise reduction in low field MRI applications. IEEE Trans Med Imaging 2022;41:1007–1016. 35089856 10.1109/TMI.2022.3147450

[20] Cooley CZ, Stockmann JP, Armstrong BD, Two-dimensional imaging in a lightweight portable MRI scanner without gradient coils. Magn Reson Med 2015;73:872–883. 24668520 10.1002/mrm.25147 PMC4257909

[21] Heiss R, Nagel AM, Laun FB, Uder M, Bickelhaupt S. Low-field magnetic resonance imaging: A new generation of breakthrough Technology in Clinical Imaging. Invest Radiol 2021;56:726–733. 34132228 10.1097/RLI.0000000000000805

[22] Campbell-Washburn AE, Ramasawmy R, Restivo MC, Opportunities in interventional and diagnostic imaging by using high-performance low-field-strength MRI. Radiology 2019;293:384–393. 31573398 10.1148/radiol.2019190452 PMC6823617

[23] Stockmann JP, Cooley CZ, Guerin B, Rosen MS, Wald LL. Transmit Array Spatial Encoding (TRASE) using broadband WURST pulses for RF spatial encoding in inhomogeneous B0 fields. J Magn Reson 2016; 268:36–48. 27155906 10.1016/j.jmr.2016.04.005 PMC4909507

[24] Lustig M, Donoho D. Compressed sensing MRI. Signal Process 2008; 25:72–82.

[25] Zhu B, Liu JZ, Cauley SF, Rosen BR, Rosen MS. Image reconstruction by domain-transform manifold learning. Nature 2018;555:487–492. 29565357 10.1038/nature25988

[26] Deoni SCL, O’Muircheartaigh J, Ljungberg E, Huentelman M, Williams SCR. Simultaneous high-resolution T 2 -weighted imaging and quantitative T 2 mapping at low magnetic field strengths using a multiple TE and multi-orientation acquisition approach. Magn Reson Med 2022;88:1273–1281. 35553454 10.1002/mrm.29273 PMC9322579

[27] Iglesias JE, Schleicher R, Laguna S, Accurate super-resolution low-field brain MRI. 2022.

[28] Iglesias JE, Billot B, Balbastre Y, Joint super-resolution and synthesis of 1 mm isotropic MP-RAGE volumes from clinical MRI exams with scans of different orientation, resolution and contrast. Neuroimage 2021;237:118206. 10.1016/j.neuroimage.2021.118206 PMC8354427 34048902

[29] Young SI, Dalca AV, Ferrante E, Golland P, Fischl B, Iglesias JE. SUD: Supervision by denoising for medical image segmentation. IEEE Trans Pattern Anal Mach Intell 2022;02952:1–16. 10.1109/TPAMI.2023.3299789 PMC12498241 37505997

[30] Arnold TC, Baldassano SN, Litt B, Stein JM. Simulated diagnostic performance of low-field MRI: Harnessing open-access datasets to evaluate novel devices. Magn Reson Imaging 2022;87:67–76. 34968700 10.1016/j.mri.2021.12.007 PMC8816889

[31] Le DBT, Sadinski M, Nacev A, Narayanan R, Kumar D. Deep learning-based method for denoising and image enhancement in low-field MRI. IST 2021 - IEEE Int Conf Imaging Syst Tech Proc. Piscataway, NJ: Institute of Electrical and Electronics Engineers Inc.; 2021.

[32] Manso Jimeno M, Ravi KS, Jin Z, Oyekunle D, Ogbole G, Geethanath S. ArtifactID: Identifying artifacts in low-field MRI of the brain using deep learning. Magn Reson Imaging 2022;89:42–48. 35176447 10.1016/j.mri.2022.02.002

[33] Moritz M, Redlich T, Günyar S, Winter L, Wulfsberg JP. On the economic value of open source hardware – Case study of an open source magnetic resonance imaging scanner. J Open Hardw 2019;3:2.

[34] Han H, Moritz R, Oberacker E, Waiczies H, Niendorf T, Winter L. Open source 3D multipurpose measurement system with submillimetre fidelity and first application in magnetic resonance. Sci Rep 2017;7: 13452. 29044156 10.1038/s41598-017-13824-z PMC5647334

[35] Guallart-Naval T, Algarín JM, Pellicer-Guridi R, Portable magnetic resonance imaging of patients indoors, outdoors and at home. arXiv 2022;12:1–11. 10.1038/s41598-022-17472-w PMC9338984 35907975

[36] Reilly TO, Webb AG, O’Reilly T, In vivo 3D brain and extremity MRI at 50 mT using a permanent magnet Halbach array. Magn Reson Med 2021;85:495–505. 32627235 10.1002/mrm.28396 PMC7689769

[37] Wald LL, McDaniel PC, Witzel T, Stockmann JP, Cooley CZ. Low-cost and portable MRI. J Magn Reson Imaging 2020;52:686–696. 31605435 10.1002/jmri.26942 PMC10644353

[38] Jimeno MM, Vaughan JT, Geethanath S. Superconducting magnet designs and MRI accessibility: A review. 2022. 10.1002/nbm.4921 36914280

[39] Klein H-M. Clinical low field strength magnetic resonance imaging. 2016.

[40] Deoni SCL, D’Sa V, Volpe A, Remote and at-home data collection: Considerations for the NIH HEALthy Brain and Cognitive Development (HBCD) study. Dev Cogn Neurosci 2022;54:101059. 10.1016/j.dcn.2022.101059 PMC8762360 35033972

[41] Motzkin JC, Newman JP, Kiehl KA, Koenigs M. Reduced prefrontal connectivity in psychopathy. J Neurosci 2011;31:17348–17357. 22131397 10.1523/JNEUROSCI.4215-11.2011 PMC3311922

[42] Nakagomi M, Kajiwara M, Matsuzaki J, Development of a small car-mounted magnetic resonance imaging system for human elbows using a 0.2 T permanent magnet. J Magn Reson 2019;304:1–6. 31063952 10.1016/j.jmr.2019.04.017

[43] Deoni SCL, Medeiros P, Deoni AT, Development of a mobile low-field MRI scanner. Sci Rep 2022;12:5690. 35383255 10.1038/s41598-022-09760-2 PMC8982311

[44] Papanicolas I, Woskie LR, Jha AK. Health care spending in the United States and other high-income countries. JAMA 2018;319: 1024–1039. 29536101 10.1001/jama.2018.1150

[45] Anzai Y, Minoshima S, Lee VS. Enhancing value of MRI: A call for action. J Magn Reson Imaging 2019;49:e40–e48. 30431676 10.1002/jmri.26239

[46] Hayashi N, Watanabe Y, Masumoto T, . Utilization of low-field MR scanners. Magn Reson Med Sci 2004;3:27–38. 16093617 10.2463/mrms.3.27

[47] van Beek EJR, Kuhl C, Anzai Y, Value of MRI in medicine: More than just another test? J Magn Reson Imaging 2019;49:e14–e25. 30145852 10.1002/jmri.26211 PMC7036752

[48] Holbrook A, Glenn H, Mahmood R, Cai Q, Kang J, Duszak R. Shorter perceived outpatient MRI wait times associated with higher patient satisfaction. J Am Coll Radiol 2016;13:505–509. 26768544 10.1016/j.jacr.2015.11.008

[49] Wood DA, Kafiabadi S, Al BA, Deep learning models for triaging hospital head MRI examinations. Med Image Anal 2022;78:102391. 10.1016/j.media.2022.102391 35183876

[50] Ogbole GI, Adeyomoye AO, Badu-Peprah A, Mensah Y, Nzeh DA. Survey of magnetic resonance imaging availability in West Africa. Pan Afr Med J 2018;30:240. 30574259 10.11604/pamj.2018.30.240.14000 PMC6295297

[51] Ogbole GI, Adeleye AO, Adeyinka AO, Ogunseyinde OA. Magnetic resonance imaging: Clinical experience with an open low-field-strength scanner in a resource challenged African state. J Neurosci Rural Pract 2012;3:137–143. 22865963 10.4103/0976-3147.98210 PMC3409982

[52] Lother S, Schiff SJ, Neuberger T, Jakob PM, Fidler F. Design of a mobile, homogeneous, and efficient electromagnet with a large field of view for neonatal low-field MRI. Magn Reson Mater Physics Biol Med 2016;29:691–698. 10.1007/s10334-016-0525-8 PMC5695548 26861046

[53] Obungoloch J, Harper JR, Consevage S, Design of a sustainable prepolarizing magnetic resonance imaging system for infant hydrocephalus. Magn Reson Mater Physics Biol Med 2018;31:665–676. 10.1007/s10334-018-0683-y PMC6135672 29644479

[54] Diehl JC, Van Doesum F, Bakker M, The embodiment of low-field MRI for the diagnosis of infant hydrocephalus in Uganda. 2020 IEEE Glob Humanit Technol Conf GHTC 2020. Piscataway, NJ: Institute of Electrical and Electronics Engineers Inc.; 2020.

[55] Natukunda F, Twongyirwe TM, Schiff SJ, Obungoloch J. Approaches in cooling of resistive coil-based low-field magnetic resonance imaging (MRI) systems for application in low resource settings. BMC Biomed Eng 2021;3:1–11. 33579373 10.1186/s42490-021-00048-6 PMC7881601

[56] Morris MA, Saboury B. Access to imaging technology in global health. Radiol Glob Heal Strateg Implementation, Appl. New York: Springer International Publishing; 2018. p 15–33.

[57] Mollura DJ, Culp MP, Pollack E, Artificial intelligence in low- and middle-income countries: Innovating global health radiology. Radiology 2020;297:513–520. 33021895 10.1148/radiol.2020201434

[58] Shen FX, Wolf SM, Bhavnani S, Emerging ethical issues raised by highly portable MRI research in remote and resource-limited international settings. Neuroimage 2021;238:118210. 10.1016/j.neuroimage.2021.118210 PMC8382487 34062266

[59] Shen FX, Wolf SM, Gonzalez RG, Garwood M. Ethical issues posed by field research using highly portable and cloud-enabled neuroimaging. Neuron 2020;105:771–775. 32135089 10.1016/j.neuron.2020.01.041 PMC8803403

[60] Feigin VL, Norrving B, Mensah GA. Global burden of stroke. Circ Res 2017;120:439–448. 28154096 10.1161/CIRCRESAHA.116.308413

[61] Grysiewicz RA, Thomas K, Pandey DK. Epidemiology of ischemic and hemorrhagic stroke: Incidence, prevalence, mortality, and risk factors. Neurol Clin 2008;26:871–895. 19026895 10.1016/j.ncl.2008.07.003

[62] Fassbender K, Balucani C, Walter S, Levine SR, Haass A, Grotta J. Streamlining of prehospital stroke management: The golden hour. Lancet Neurol 2013;12:585–596. 23684084 10.1016/S1474-4422(13)70100-5

[63] Nogueira RG, Jadhav AP, Haussen DC, Thrombectomy 6 to 24 hours after stroke with a mismatch between deficit and infarct. N Engl J Med 2018;378:11–21. 29129157 10.1056/NEJMoa1706442

[64] Easton JD, Saver JL, Albers GW, Definition and evaluation of transient ischemic attack: A scientific statement for healthcare professionals from the American heart association/American stroke association stroke council; council on cardiovascular surgery and anesthesia; council on cardiovascular radiology and intervention; council on cardiovascular nursing; and the interdisciplinary council on peripheral vascular disease. Stroke 2009;40:2276–2293. 19423857 10.1161/STROKEAHA.108.192218

[65] Schellinger PD, Bryan RN, Caplan LR, Evidence-based guideline: The role of diffusion and perfusion MRI for the diagnosis of acute ischemic stroke: Report of the therapeutics and technology assessment subcommittee of the american academy of neurology. Neurology 2010;75:177–185. 20625171 10.1212/WNL.0b013e3181e7c9dd PMC2905927

[66] Jonathan AE, Brazzelli MG, Warach S, Correspondence: Evidence-based guideline: the role of diffusion and perfusion MRI for the diagnosis of acute ischemic stroke: report of the Therapeutics and Technology Subcommittee of the American Academy of Neurology. Neurology 2011;76(23):2036–2038. 10.1212/WNL.0b013e318219a0b4 21646634

[67] Bhat SS, Fernandes TT, Poojar P, Low-field MRI of stroke: Challenges and opportunities. J Magn Reson Imaging 2021;54:372–390. 32827173 10.1002/jmri.27324

[68] Merino JG, Warach S. Imaging of acute stroke. Nat Rev Neurol 2010; 6:560–571. 20842186 10.1038/nrneurol.2010.129

[69] Hori M, Hagiwara A, Goto M, Wada A, Aoki S. Low-field magnetic resonance imaging: Its history and renaissance. Invest Radiol 2021;56: 669–679. 34292257 10.1097/RLI.0000000000000810 PMC8505165

[70] Terada H, Gomi T, Harada H, Development of diffusion-weighted image using a 0.3T open MRI. J Neuroradiol 2006;33:57–61. 16528207 10.1016/s0150-9861(06)77229-7

[71] Hori M, Aoki S, Okubo T, Ishigame K, Kumagai H, Araki T. Line-scan diffusion tensor MR imaging at 0.2 T: Feasibility study. J Magn Reson Imaging 2005;22:794–798. 16270295 10.1002/jmri.20440

[72] Sheth KN, Mazurek MH, Yuen MM, Assessment of brain injury using portable, low-field magnetic resonance imaging at the bedside of critically ill patients. JAMA Neurol 2021;78:41–47. 10.1001/jamaneurol.2020.3263 PMC7489395 32897296

[73] Mazurek MH, Cahn BA, Yuen MM, Portable, bedside, low-field magnetic resonance imaging for evaluation of intracerebral hemorrhage. Nat Commun 2021;12:5119. 34433813 10.1038/s41467-021-25441-6 PMC8387402

[74] Mills TT. FDA Clearance K192002 - Lucy Point-of-Care Magnetic Resonance Imaging Device. FDA. 2020.

[75] Turpin J, Unadkat P, Thomas J, Portable magnetic resonance imaging for ICU patients. Crit Care Explor 2020;2:e0306. 33381764 10.1097/CCE.0000000000000306 PMC7769347

[76] Sheth KN, Yuen MM, Mazurek MH, Bedside detection of intracranial midline shift using portable magnetic resonance imaging. Sci Rep 2022;12:67. 34996970 10.1038/s41598-021-03892-7 PMC8742125

[77] Kundu P, Sadegh S, Salehi M, Cahn BA, Mazurek MH. Point-of-care MRI with artificial intelligence to measure midline shift in acute stroke follow-up. 2022.

[78] Saver JL. Time is brain - quantified. Stroke 2006;37:263–266. 16339467 10.1161/01.STR.0000196957.55928.ab

[79] Parker SA, Bowry R, Wu TC, Establishing the first Mobile stroke unit in the United States. Stroke 2015;46:1384–1391. 25782464 10.1161/STROKEAHA.114.007993

[80] Grotta JC, Yamal J-M, Parker SA, Prospective, multicenter, controlled trial of mobile stroke units. N Engl J Med 2021;385:971–981. 34496173 10.1056/NEJMoa2103879

[81] Prabhat AM, Crawford AL, Mazurek MH, Methodology for low-field, portable magnetic resonance neuroimaging at the bedside. Front Neurol 2021;12:1–12. 10.3389/fneur.2021.760321 PMC8703196 34956049

[82] Albers GW, Marks MP, Kemp S, Thrombectomy for stroke at 6 to 16 hours with selection by perfusion imaging. N Engl J Med 2018;378:708–718. 29364767 10.1056/NEJMoa1713973 PMC6590673

[83] Waddington DEJ, Boele T, Maschmeyer R, Kuncic Z, Rosen MS. High-sensitivity in vivo contrast for ultra-low field magnetic resonance imaging using superparamagnetic iron oxide nanoparticles. Sci Adv 2020; 6:1–10. 10.1126/sciadv.abb0998 PMC7367688 32733998

[84] Neuwelt EA, Varallyay CG, Manninger S, The potential of ferumoxytol nanoparticle magnetic resonance imaging, perfusion, and angiography in central nervous system malignancy: A pilot study. Neurosurgery 2007;60:601–611. 17415196 10.1227/01.NEU.0000255350.71700.37

[85] Toth GB, Varallyay CG, Horvath A, Current and potential imaging applications of ferumoxytol for magnetic resonance imaging. Kidney Int 2017;92:47–66. 28434822 10.1016/j.kint.2016.12.037 PMC5505659

[86] Varallyay CG, Toth GB, Fu R, What does the boxed warning tell us? Safe practice of using ferumoxytol as an MRI contrast agent. Am J Neuroradiol 2017;38:1297–1302. 28495944 10.3174/ajnr.A5188 PMC5509484

[87] Arnold TC, By S, Dyvorne H, In-vivo ferumoxytol imaging and T1/T2 characterization at 64mT. Proc Intl Soc Mag Reson Med 2021; 29:1251.

[88] Campbell-Washburn AE, Jiang Y, Körzdörfer G, Nittka M, Griswold MA. Feasibility of MR fingerprinting using a high-performance 0.55 T MRI system. Magn Reson Imaging 2021;81:88–93. 34116134 10.1016/j.mri.2021.06.002 PMC8749356

[89] Runge VM, Heverhagen JT. Advocating the development of next-generation, advanced-design low-field magnetic resonance systems. Invest Radiol 2020;55:747–753. 33156083 10.1097/RLI.0000000000000703

[90] Van Speybroeck CDE, O’Reilly T, Teeuwisse W, Arnold PM, Webb AG. Characterization of displacement forces and image artifacts in the presence of passive medical implants in low-field (<100 mT) permanent magnet-based MRI systems, and comparisons with clinical MRI systems. Phys Medica 2021;84:116–124. 10.1016/j.ejmp.2021.04.003 33894581

[91] World Health Organization. Neurological disorders: Public health challenges. 2006.

[92] Feigin VL, Abajobir AA, Abate KH, Global, regional, and national burden of neurological disorders during 1990–2015: A systematic analysis for the Global Burden of Disease Study 2015. Lancet Neurol 2017;16:877–897. 28931491 10.1016/S1474-4422(17)30299-5 PMC5641502

[93] Obungoloch J. Development of ultra low field magnetic resonance imaging for diagnosis of hydrocephalus in developing countries. Ann Arbor, MI: The Pennsylvania State University; 2017.

[94] Arnold TC, By S, Welch EB, Monitoring hydrocephalus patients using portable, low-field MRI. Radiol Soc North Am Sci Assem Annu Meet. Chicago, IL: Chicago; 2021.

[95] Mateen FJ, Cooley CZ, Stockmann JP, Rice DR, Vogel AC, Wald LL. Low-field portable brain MRI in CNS demyelinating disease. Mult Scler Relat Disord 2021;51:102903. 10.1016/j.msard.2021.102903 33780808

[96] Arnold TC, Tu D, Okar SV, Sensitivity of portable low-field magnetic resonance imaging for multiple sclerosis lesions. NeuroImage Clin 2022;35:103101. 10.1016/j.nicl.2022.103101 PMC9421456 35792417

[97] Cuingnet R, Gerardin E, Tessieras J, Automatic classification of patients with Alzheimer’s disease from structural MRI: A comparison of ten methods using the ADNI database. Neuroimage 2011;56: 766–781. 20542124 10.1016/j.neuroimage.2010.06.013

[98] Abi-Dargham A, Horga G. The search for imaging biomarkers in psychiatric disorders. Nat Med 2016;22:1248–1255. 27783066 10.1038/nm.4190

[99] Möller C, Vrenken H, Jiskoot L, Different patterns of gray matter atrophy in early- and late-onset Alzheimer’s disease. Neurobiol Aging 2013;34:2014–2022. 23561509 10.1016/j.neurobiolaging.2013.02.013

[100] Roman GC, Tatemichi TK, Erkinjuntti T, Vascular dementia: Diagnostic criteria for research studies: Report of the ninds-airen international workshop. Neurology 1993;43:250–260. 8094895 10.1212/wnl.43.2.250

[101] Hill DLG, Schwarz AJ, Isaac M, Coalition against major diseases/European Medicines Agency biomarker qualification of hippocampal volume for enrichment of clinical trials in predementia stages of Alzheimer’s disease. Alzheimers Dement 2014;10:421429.e3. 10.1016/j.jalz.2013.07.003 24985687

[102] Deoni SCL, Bruchhage MMK, Beauchemin J, Accessible pediatric neuroimaging using a low field strength MRI scanner. Neuroimage 2021;238:118273. 10.1016/j.neuroimage.2021.118273 34146712

[103] Cali RJ, Freeman HJ, Billot B, Synthesis of high-resolution research-quality MRI data from clinical MRI data in patients with COVID-19. medRxiv 2021;21266090:1–16.

[104] Kirkman MA. The role of imaging in the development of neurosurgery. J Clin Neurosci 2015;22:55–61. 25150767 10.1016/j.jocn.2014.05.024

[105] Kesserwan MA, Shakil H, Lannon M, Frame-based versus frameless stereotactic brain biopsies: A systematic review and meta-analysis. Surg Neurol Int 2021;12:52. 33654555 10.25259/SNI_824_2020 PMC7911151

[106] Orringer DA, Golby A, Jolesz F. Neuronavigation in the surgical management of brain tumors: Current and future trends. Expert Rev Med Devices 2012;9:491–500. 23116076 10.1586/erd.12.42 PMC3563325

[107] Black PM, Moriarty T, Alexander E, Development and implementation of intraoperative magnetic resonance imaging and its neurosurgical applications. Neurosurgery 1997;41:831–845. 9316044 10.1097/00006123-199710000-00013

[108] Tronnier VM, Wirtz CR, Knauth M, Intraoperative diagnostic and interventional magnetic resonance imaging in neurosurgery. Neurosurgery 1997;40:891–902. 9149246 10.1097/00006123-199705000-00001

[109] Sutherland GR, Kaibara T, Louw D, Hoult DI, Tomanek B, Saunders J. A mobile high-field magnetic resonance system for neurosurgery. J Neurosurg 1999;91:804–813. 10541238 10.3171/jns.1999.91.5.0804

[110] Hadani M, Spiegelman R, Feldman Z, Berkenstadt H, Ram Z. Novel, compact, intraoperative magnetic resonance imaging-guided system for conventional neurosurgical operating rooms. Neurosurgery 2001; 48:799–809. 11322440 10.1097/00006123-200104000-00021

[111] Hlavac M, Wirtz CR, Halatsch ME. Intraoperative magnetresonanztomographie. HNO 2017;65:25–29. 27670420 10.1007/s00106-016-0240-9

[112] Senft C, Bink A, Franz K, Vatter H, Gasser T, Seifert V. Intraoperative MRI guidance and extent of resection in glioma surgery: A randomised, controlled trial. Lancet Oncol 2011;12:997–1003. 21868284 10.1016/S1470-2045(11)70196-6

[113] Schneider JP, Trantakis C, Rubach M, Intraoperative MRI to guide the resection of primary supratentorial glioblastoma multiforme - A quantitative radiological analysis. Neuroradiology 2005;47: 489–500. 15951997 10.1007/s00234-005-1397-1

[114] Li P, Qian R, Niu C, Fu X. Impact of intraoperative MRI-guided resection on resection and survival in patient with gliomas: A meta-analysis. Curr Med Res Opin 2017;33:621–630. 28008781 10.1080/03007995.2016.1275935

[115] Nimsky C, Ganslandt O, Hastreiter P, Fahlbusch R. Intraoperative compensation for brain shift. Surg Neurol 2001;56:357–364. 11755962 10.1016/s0090-3019(01)00628-0

[116] Lewin JS, Nour SG, Duerk JL. Magnetic resonance image-guided biopsy and aspiration. Top Magn Reson Imaging 2000;11:173–183. 11145209 10.1097/00002142-200006000-00003

[117] Gasser T, Ganslandt O, Sandalcioglu E, Stolke D, Fahlbusch R, Nimsky C. Intraoperative functional MRI: Implementation and preliminary experience. Neuroimage 2005;26:685–693. 15955478 10.1016/j.neuroimage.2005.02.022

[118] Nimsky C. Intraoperative acquisition of fMRI and DTI. Neurosurg Clin N Am 2011;22:269–277. 21435576 10.1016/j.nec.2010.11.005

[119] Jethwa PR, Barrese JC, Gowda A, Shetty A, Danish SF. Magnetic resonance thermometry-guided laser-induced thermal therapy for intracranial neoplasms: Initial experience. Neurosurgery 2012;71(Suppl 1):133. 22653396 10.1227/NEU.0b013e31826101d4

[120] Englman C, Malpas CB, Harvey AS, Maixner WJ, Yang JYM. Intraoperative magnetic resonance imaging in epilepsy surgery: A systematic review and meta-analysis. J Clin Neurosci 2021;91:1–8. 34373012 10.1016/j.jocn.2021.06.035

[121] Hall WA, Truwit CL. Intraoperative MR-guided neurosurgery. J Magn Reson Imaging 2008;27:368–375. 18183585 10.1002/jmri.21273

[122] Livne O, Harel R, Hadani M, Spiegelmann R, Feldman Z, Cohen ZR. Intraoperative magnetic resonance imaging for resection of intra-axial brain lesions: A decade of experience using low-field magnetic resonance imaging, polestar n-10, 20, 30 systems. World Neurosurg 2014; 82:770–776. 24518885 10.1016/j.wneu.2014.02.004

[123] Bellut D, Hlavica M, Schmid C, Bernays RL. Intraoperative magnetic resonance imaging-assisted transsphenoidal pituitary surgery in patients with acromegaly. Neurosurg Focus 2010;29:1–9. 20887134 10.3171/2010.7.FOCUS10164

[124] Hlavica M, Bellut D, Lemm D, Schmid C, Bernays RL. Impact of ultralow-field intraoperative magnetic resonance imaging on extent of resection and frequency of tumor recurrence in 104 surgically treated nonfunctioning pituitary adenomas. World Neurosurg 2013;79: 99–109. 23043996 10.1016/j.wneu.2012.05.032

[125] Kim EH, Oh MC, Kim SH. Application of low-field intraoperative magnetic resonance imaging in transsphenoidal surgery for pituitary adenomas: Technical points to improve the visibility of the tumor resection margin. Acta Neurochir 2013;155:485–493. 23318686 10.1007/s00701-012-1608-6

[126] Wu J-S, Shou X-F, Yao C-J, Transsphenoidal pituitary macroadenomas resection guided by PoleStar N20 low-field intraoperative magnetic resonance imaging. Neurosurgery 2009;65: 63–71. 19574826 10.1227/01.NEU.0000348549.26832.51

[127] Ginat DT, Swearingen B, Curry W, Cahill D, Madsen J, Schaefer PW. 3 tesla intraoperative MRI for brain tumor surgery. J Magn Reson Imaging 2014;39:1357–1365. 24921066 10.1002/jmri.24380

[128] Pamir MN, Özduman K, Dinçer A, Yildiz E, Peker S, Özek MM. First intraoperative, shared-resource, ultrahigh-field 3-tesla magnetic resonance imaging system and its application in low-grade glioma resection: Clinical article. J Neurosurg 2010;112:57–69. 19480544 10.3171/2009.3.JNS081139

[129] Hatiboglu MA, Weinberg JS, Suki D, Impact of intraoperative high-field magnetic resonance imaging guidance on glioma surgery. Neurosurgery 2009;64:1073–1081. 19487886 10.1227/01.NEU.0000345647.58219.07

[130] Hall WA, Kowalik K, Liu H, Truwit CL, Kucharczyk J. Costs and benefits of intraoperative MR-guided brain tumor resection. Acta Neurochir Suppl. New York: Springer Wien; 2003. p 137–142. 10.1007/978-3-7091-6043-5_19 12570149

[131] Makary M, Chiocca EA, Erminy N, Clinical and economic outcomes of low-field intraoperative MRI-guided tumor resection neurosurgery. J Magn Reson Imaging 2011;34:1022–1030. 22002753 10.1002/jmri.22739

[132] Kucharczyk W, Bernstein M. Do the benefits of image guidance in neurosurgery justify the costs? From stereotaxy to intraoperative MR. AJNR Am J Neuroradiol 1997;18:1855–1859. 9403441 PMC8337367

[133] Amin EK, Campbell-Washburn A, Ratnayaka K. MRI-guided cardiac catheterization in congenital heart disease: How to get started. Curr Cardiol Rep 2022;24:419–429. 35107702 10.1007/s11886-022-01659-8 PMC8979923

[134] Stainsby JA, Bindseil GA, Connell IR, Imaging at 0.5 T with high-performance system components system components. Proc Intl Soc Mag Reson Med 2019;27:1194.

[135] Stainsby JA, Harris CT, Curtis AT, Beatty PJ, Wiens CN. Diffusion tractography at 0.5T: Comparison to 1.5T. Proc Intl Soc Mag Reson Med 2020;28:4544.

[136] Connell IR, Panther A, Chronik BA. Specific absorption rate in headonly mid-field scanner: Comparisons to 1.5 T and 3 T. Proc Intl Soc Mag Reson Med 2019;27:4167.

[137] Mutic S, Dempsey JF. The ViewRay system: Magnetic resonanceguided and controlled radiotherapy. Semin Radiat Oncol 2014;24: 196–199. 24931092 10.1016/j.semradonc.2014.02.008

[138] Nasri J, Wagaskar VG, Parekha S, Office-based, point-of-care, low-field MRI system to guide prostate interventions: Recent developments. EMJ Urol 2021;9:83–90.

[139] Chiragzada S, Hellman E, Michael D, Narayanan R, Nacev A, Kumar D. Initial phantom studies for an office-based low-field MR system for prostate biopsy. Int J Comput Assist Radiol Surg 2021;16: 741–748. 33891253 10.1007/s11548-021-02364-7 PMC8134310

[140] Scheinfeld MH, Moon JY, Fagan MJ, Davoudzadeh R, Wang D, Taragin BH. MRI usage in a pediatric emergency department: An analysis of usage and usage trends over 5 years. Pediatr Radiol 2017; 47:327–332. 28083700 10.1007/s00247-016-3764-y

[141] Raschle N, Zuk J, Ortiz-Mantilla S, Pediatric neuroimaging in early childhood and infancy: Challenges and practical guidelines. Ann N Y Acad Sci 2012;1252:43–50. 22524338 10.1111/j.1749-6632.2012.06457.x PMC3499030

[142] Wood JR, Pedersen RC, Rooks VJ. Neuroimaging for the primary care provider: A review of modalities, indications, and pitfalls. Pediatr Clin North Am 2021;68:715–725. 34247704 10.1016/j.pcl.2021.04.014

[143] Health risks from exposure to low levels of ionizing radiation: BEIR VII Phase 2 - National Research Council, Division on Earth and Life Studies, Board on Radiation Effects Research, Committee to Assess Health Risks from Exposure to Low Levels of Ionizing Radiation - Google Books. Available from: https://books.google.com/books?hl=en&lr=&id=H4J3Ns_3lUIC&oi=fnd&pg=PR1&dq=Health+risks+from+exposure+to+low+levels+of+ionizing+radiation:+BEIR+VII+phase+2&ots=mGDZXvt7MT&sig=ItWdT-BEbTU3Z0iHnl9Xb3pnEnE#v=onepa ge&q=Healthrisksfromexposuretolowlevelsofionizingradiation%3AB EIRVIIphase2&f=false

[144] Holmedal LJ, Friberg EG, Børretzen I, Olerud H, Lægreid L, Rosendahl K. Radiation doses to children with shunt-treated hydrocephalus. Pediatr Radiol 2007;37:1209–1215. 17926028 10.1007/s00247-007-0625-8

[145] Muhogora WE, Ahmed NA, AlSuwaidi JS, Paediatric CT examinations in 19 developing countries: Frequency and radiation dose. Radiat Prot Dosimetry 2010;140:49–58. 20154022 10.1093/rpd/ncq015

[146] Barkovich MJ, Li Y, Desikan RS, Barkovich AJ, Xu D. Challenges in pediatric neuroimaging. Neuroimage 2019;185:793–801. 29684645 10.1016/j.neuroimage.2018.04.044 PMC6197938

[147] Rupprecht T, Kuth R, Böwing B, Gerling S, Wagner M, Rascher W. Sedation and monitoring of paediatric patients undergoing open low-field MRI. Acta Paediatr 2007;89:1077–1081. 10.1080/713794566 11071088

[148] Wagner M, Böwing B, Kuth R, Deimling M, Rascher W, Rupprecht T. Low field thoracic MRI - A fast and radiation free routine imaging modality in children. Magn Reson Imaging 2001;19:975–983. 11595369 10.1016/s0730-725x(01)00417-9

[149] Nimsky C, Ganslandt O, Gralla J, Buchfelder M, Fahlbusch R. Intraoperative low-field magnetic resonance imaging in pediatric neurosurgery. Pediatr Neurosurg 2003;38:83–89. 12566841 10.1159/000068046

[150] Whitby EH, Paley MN, Smith MF, Sprigg A, Woodhouse N, Griffiths PD. Low field strength magnetic resonance imaging of the neonatal brain. Arch Dis Child Fetal Neonatal Ed 2003;88:F203F208. 10.1136/fn.88.3.F203 PMC1721544 12719393

[151] Thiim KR, Singh E, Mukundan S, Clinical experience with an in-NICU magnetic resonance imaging system. J Perinatol 2022;42: 873–879. 35459908 10.1038/s41372-022-01387-5 PMC9026005

[152] Fischer HW, Rinck PA, van Haverbeke Y, Muller RN. Nuclear relaxation of human brain gray and white matter: Analysis of field dependence and implications for MRI. Magn Reson Med 1990;16: 317–334. 2266850 10.1002/mrm.1910160212

[153] Chetcuti K, Chilingulo C, Goyal MS, Implementation of a low-field portable MRI scanner in a resource-constrained environment: Our experience in Malawi. Am J Neuroradiol 2022;43:670–674. 35450856 10.3174/ajnr.A7494 PMC9089250

[154] Tavernier T, Cotten A. High- versus low-field MR imaging. Radiol Clin North Am 2005;43:673–681. 15893530 10.1016/j.rcl.2005.02.001

[155] Baker MA, MacKay S. Please be upstanding – A narrative review of evidence comparing upright to supine lumbar spine MRI. Radiography 2021;27:721–726. 33268049 10.1016/j.radi.2020.11.003

[156] Vellet AD, Lee DH, Munk PL, Anterior cruciate ligament tear: Prospective evaluation of diagnostic accuracy of middle- and highfield-strength MR imaging at 1.5 and 0.5 T. Radiology 1995;197: 826–830. 7480763 10.1148/radiology.197.3.7480763

[157] Merl T, Scholz M, Gerhardt P, Results of a prospective multicenter study for evaluation of the diagnostic quality of an open whole-body low-filed MRI unit. A comparison with high-field MRI measured by the applicable gold standard. Eur J Radiol 1999;30:43–53. 10389012 10.1016/s0720-048x(98)00134-x

[158] Zlatkin MB, Hoffman C, Shellock FG. Assessment of the rotator cuff and glenoid labrum using an extremity MR system: MR results compared to surgical findings from a multi-center study. J Magn Reson Imaging 2004;19:623–631. 15112313 10.1002/jmri.20040

[159] Magee T, Shapiro M, Williams D. Comparison of high-field-strength versus low-field-strength MRI of the shoulder. Am J Roentgenol 2003; 181:1211–1215. 14573405 10.2214/ajr.181.5.1811211

[160] Thomsen HS, Larsen L, Chabanova E, Moller JM. Open low-fieldstrength MRI of the shoulder is not so bad. Am J Roentgenol 2004; 182:1601–1602. 15150022 10.2214/ajr.182.6.1821601

[161] Ghazinoor S, Iii JVC, Crowley C. Low-field musculoskeletal MRI. J Magn Reson Imaging 2007;244:234–244. 10.1002/jmri.20854 17260396

[162] Oei EHG, Nikken JJ, Verstijnen ACM, Ginai AZ, Hunink MGM. MR imaging of the menisci and cruciate ligaments: A systematic review. Radiology 2003;226:837–848. 12601211 10.1148/radiol.2263011892

[163] Steinborn M, Heuck A, Jessel C, Bonel H, Reiser M. Magnetic resonance imaging of lateral epicondylitis of the elbow with a 0.2-T dedicated system. Eur Radiol 1999;9:1376–1380. 10460377 10.1007/s003300050851

[164] Breitenseher MJ, Trattnig S, Gäbler C, Radiologically occult scaphoid and wrist fractures. Preliminary results in comparison of 0.2-T and 1.0-T units. Radiologe 1997;37:812–818. 9454275 10.1007/s001170050287

[165] Verhoek G, Zanetti M, Duewell S, Zollinger H, Hodler J. MRI of the foot and ankle: Diagnostic performance and patient acceptance of a dedicated low field MR scanner. J Magn Reson Imaging 1998;8:711–716. 9626891 10.1002/jmri.1880080330

[166] Watchmaker JM, Xia D, Dayan E, Point-of-care knee imaging using a 64mT portable MRI scanner: First results. ISMRM Low-Field MRI Workshop. 2022.

[167] Khodarahmi I, Brinkmann IM, Lin DJ, New-generation low-field magnetic resonance imaging of hip arthroplasty implants using slice encoding for metal artifact correction. Invest Radiol 2022;57:1–10. 35239614 10.1097/RLI.0000000000000866 PMC9363001

[168] Guerini H, Omoumi P, Guichoux F, Fat suppression with Dixon techniques in musculoskeletal magnetic resonance imaging: A pictorial review. Semin Musculoskelet Radiol 2015;19:335–347. 26583362 10.1055/s-0035-1565913

[169] Delfaut EM, Beltran J, Johnson G, Rousseau J, Marchandise X, Cotten A. Fat suppression in MR imaging: Techniques and pitfalls. Radiographics 1999;19:373–382. 10194785 10.1148/radiographics.19.2.g99mr03373

[170] Bellisari FC, Bruno F, Monti R, Diagnostic performance of DIXON sequences on low-field scanner for the evaluation of knee joint pathology. Acta Biomed 2021;92:18–22. 10.23750/abm.v92iS5.11870 PMC8477066 34505845

[171] Raman S, Gold GE, Rosen MS, Sveinsson B. Automatic estimation of knee effusion from limited MRI data. Sci Rep 2022;12:3155. 35210490 10.1038/s41598-022-07092-9 PMC8873489

[172] Dam EB, Lillholm M, Marques J, Nielsen M. Automatic segmentation of high- and low-field knee MRIs using knee image quantification with data from the osteoarthritis initiative. J Med Imaging 2015;2:024001. 10.1117/1.JMI.2.2.024001 PMC4478858 26158096

